# *Yijung-tang* improves thermogenesis and reduces inflammation associated with gut microbiota in hypothyroid rats

**DOI:** 10.1038/s41522-023-00396-2

**Published:** 2023-06-03

**Authors:** Saeid Khakisahneh, Xue-Ying Zhang, Song-Yi Han, Eun-Ji Song, Young-Do Nam, Hojun Kim

**Affiliations:** 1 Department of Rehabilitation Medicine of Korean Medicine, Dongguk University, 814 Siksa-dong, Ilsandong-gu, Goyang-si 10326 Republic of Korea; 2 State Key Laboratory of Integrated Management of Pest Insects and Rodents, Institute of Zoology, Chinese Academy of Sciences, Beijing, 100101 China; 3 CAS Center for Excellence in Biotic Interactions, University of Chinese Academy of Sciences, Beijing, 100049 China; 4 Research Group of Gut Microbiome, Korea Food Research Institute, Wanju-gun, 245 Republic of Korea; 5 Department of Food Biotechnology, Korea University of Science and Technology, Wanju, Republic of Korea

**Keywords:** Applied microbiology, Biofilms

## Abstract

Currently, considerable attention is focused on exploring the potential relationship between herbal medicine (HM) and the gut microbiome in terms of thermoregulation, which is an important aspect of human health, in modern system biology. However, our knowledge of the mechanisms of HM in thermoregulation is inadequate. Here, we demonstrate that the canonical herbal formula, *Yijung-tang* (YJT), protects against hypothermia, hyperinflammation, and intestinal microbiota dysbiosis in PTU-induced hypothyroid rats. Notably, these properties were associated with alterations in the gut microbiota and signaling crosstalk between the thermoregulatory and inflammatory mediators in the small intestine and brown adipose tissue (BAT). In contrast to the conventional drug L-thyroxine for curing hypothyroidism, YJT has an efficacy for attenuating systematic inflammatory responses, related with depression in intestinal TLR4 and Nod2/Pglyrp1 signaling pathways. Our findings suggest that YJT could promote BAT thermogenesis and prevent systemic inflammation in PTU-induced hypothyroid rats, which was associated with its prebiotic effect on modulating of the gut microbiota and gene expression with relevance in the enteroendocrine function and innate immune systems. These findings may strengthen the rationale of the microbiota–gut–BAT axis for a paradigm shift to enable holobiont-centric medicine.

## Introduction

Thermoregulation is a key feature in the maintenance of homeostasis that allows organ processes to work effectively, and its close connection with thyroid hormones is well-documented^1,2^. With an increase in metabolic diseases in recent years, thyroid dysfunctions, especially hypothyroidism, represent the most common endocrine disorders and are a major healthcare issue that affects ~4–10% of the population worldwide^3,4^. The conventional treatment of hypothyroidism generates adverse drug events, high treatment costs, and compliance issues, which prompts scientists to identify alternative potential strategies for treating chronic physiological disorders. In this context, herbal medicine (HM), such as traditional Chinese and Korean medicine, has been extensively studied for its potential in treating hypothyroidism, based on a range of unique theories and significant clinical experience^5–7^.

Hypothyroidism exhibits diminishing energy metabolism, and thus drugs with warm properties because of their potential impacts on increasing energy metabolism have been used for its treatment^5^. *Yijung-tang* (YJT), also known as *Li-Zhong-Tang*, is a canonical Chinese herbal formula with warm properties that was first described in the Treatise on Febrile and Miscellaneous Diseases by *Zhongjing Zhang* 1800 years ago. It is frequently used to alleviate the symptoms of hypothyroidism, and exhibits anti-oxidative and immunomodulatory effects^8^. YJT comprises the following herbs: *Zingiber officinale Rose* (Family: *Zingiberaceae*) rhizome (9 g), *Atractylodes macrocephala Koidz* (Family: *Asteraceae*) rhizome (9 g), *Codonopsis pilosula* (Franch.) Nannf. (Family: *Campanulaceae*) root (9 g), and *Glycyrrhiza uralensis Fisch*. Ex DC. (Family: *Fabaceae*) rhizome (9 g)^8^. However, the pharmacology and mechanisms of its warm properties have not yet been investigated.

As a crucial factor in the physiology of mammals, the microbiome has emerged as a novel research field that constitutes an integral part of health. Recent technological advancements with the launching of various international projects on the human gut microbiome have allowed an extensive study and hotspot research between chronic diseases and the gut microbiome related to human health^9,10^. Previous studies have identified the association between the gut microbiome and thyroid pathophysiology, and demonstrated changes in the concentration of thyroid hormones through different ways including an alteration in the composition of intestinal bacteria as well as the effect on iodothyronine deiodinases (Dios)^11,12^. Moreover, the thyroid–gut axis has been proposed to impact the entire metabolism by the recycling of thyroid hormones^13^. Meanwhile, as the gut microbiota are structurally dynamic over time and plastic under different conditions, and since one of the characteristics of HM is that it can be administered orally, HM will interact with the intestinal flora inevitably and will help to support the homeostasis of the gut microbiome. Therefore, the gut microbiome as a channel can exert pharmacological effects of HM on the host^14,15^. YJT contains prebiotic polysaccharides that can selectively stimulate the growth and activity of beneficial bacteria in the gut, and as a food source for these bacteria, promote the production of short-chain fatty acids (SCFAs) to support gut health and overall wellness^16,17^. This evidence led us to the hypothesis that YJZ might regulate thermogenesis and inflammation through reprogramming of the gut microbiota and multiple signaling pathways to alleviate the symptoms of hypothyroidism.

By establishing the hypothyroidism model and performing 16 S rRNA gene sequencing, we aimed to demonstrate that YJT elevates the body temperature (T_b_), diminishes systemic inflammation, and reverses hypothyroidism-associated intestinal microbiota dysbiosis. The symptoms of hypothyroidism could be induced in wild-type rats with antibiotic treatment by a cecal microbiota transfer (CMT) from hypothyroid individuals, and these symptoms are absent in the recipients by CMT from YJT-treated hypothyroidism. We further demonstrated that YJT stimulates thermogenesis of brown adipose tissue (BAT) and prevents systemic inflammation through reprogramming of the gut microbiota and related multiple signaling pathways.

## Results

### YJT alleviates hypothyroidism-induced hypothermia and systemic inflammation

**Fig. 1:**
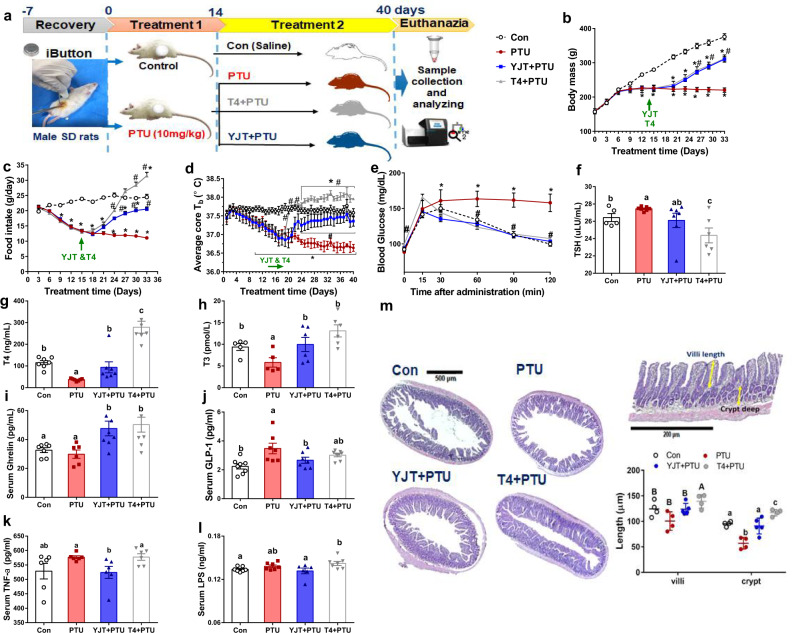
Effects of *Yijung-tang* (YJT) and L-thyroxine (T4) in metabolic phenotypes, serum metabolites, and histology in hypothyroid rats. **a** Schematic overview of the experimental design; **b** body mass; **c** food intake; **d** average core body temperature (*T*_b_) during the experiment (*n* = 5 per group). **e** Glucose metabolism was assessed via an oral glucose tolerance test. **f**–**h**The levels of thyroid stimulating hormone (TSH), thyroxine (T4), and tri-iodothyronine (T3). **i**–**l** Serum ghrelin, glucagon-like peptide-1 (GLP-1), tumor necrosis factor-α (TNF-α), and lipopolysaccharides (LPS). **m** Histology assay for the ileum by hematoxylin and eosin for four experimental groups. Data are presented as means ± SEM (*n* = 7–8 per group). One-way ANOVA or ANCOVA (food intake) followed by post hoc LSD test. *P < 0.05, versus control, ^#^*P* < 0.05 versus PTU. Different letters above the columns indicate significant differences. Con, control group that received only saline; PTU, the rats that received 10 mg/kg/body propylthiouracil (PTU); YJT + PTU, the rats that received 2.1 g/kg YJT and 10 mg/kg/body PTU; T4 + PTU, the rats that were treated with 0.5 mg/kg L-thyroxine and 10 mg/kg PTU.

Whether YJT could improve T_b_ and prevent systemic inflammation was tested in a propylthiouracil (PTU)-induced hypothyroidism rat model, with L-thyroxine (T4) as a reference drug (Fig. 1a). PTU treatment reduced the increase of body mass (Fig. 1b), and induced a 50% reduction in food intake (Fig. 1c) and a > 1 °C decrease in the average T_b_ (Fig. 1d, Supplementary Figure 1a) compared with the control. Both YJT and T4 inhibited PTU-induced loss in body mass, food intake, and T_b_ from approximately 9 days after administration (Fig. 1b–d). Moreover, YJT treatment led to a recovery in these metabolic parameters, whereas long-term T4 treatment induced hyperphagia and hyperthermia. Both YJT and T4 recovered glucose tolerance that was disrupted by PTU (Fig. 1e). As predicted, a significant increase in the serum thyroid stimulating hormone (TSH) levels and decreases in T4 and tri-iodothyronine (T3) levels were detected in rats with PTU-induced hypothyroidism compared with controls (Fig. 1f–h). YJT reversed the serum T4 and T3 levels to the control levels (Fig. 1f–h). Although PTU did not affect the serum ghrelin levels (anorexigenic hormone, Fig. 1i), it increased the secretion of glucagon-like peptide-1 (GLP-1) (an anorexigenic hormone, Fig. 1j), resulting in reduced food intake in PTU-treated rats. Both YJT and T4 treatment stimulated ghrelin secretion, and YJT, rather than T4, prevented PTU-induced increases of serum GLP-1 levels (Fig. 1i, j). The circulating inflammatory markers such as tumor necrosis factor-α (TNF-α) and lipopolysaccharides (LPS) were enhanced both in the PTU-treated and T4 + PTU-treated groups, whereas YJT prevented PTU-induced systemic inflammation (Fig. 1k, l). Moreover, histology of the ileum indicated that hypothyroidism disturbed the intestinal barrier and absorption indicated by decreased villi length and crypt depth, while both YJT and T4 treatment reversed these variables (Fig. 1m). T4 treatment caused a significant increase in the length of the small intestine and the mass of some organs including the liver, heart, and spleen (Supplementary Figure 1b, c).

### YJT regulates intestinal hormones and inflammatory cytokines

**Fig. 2:**
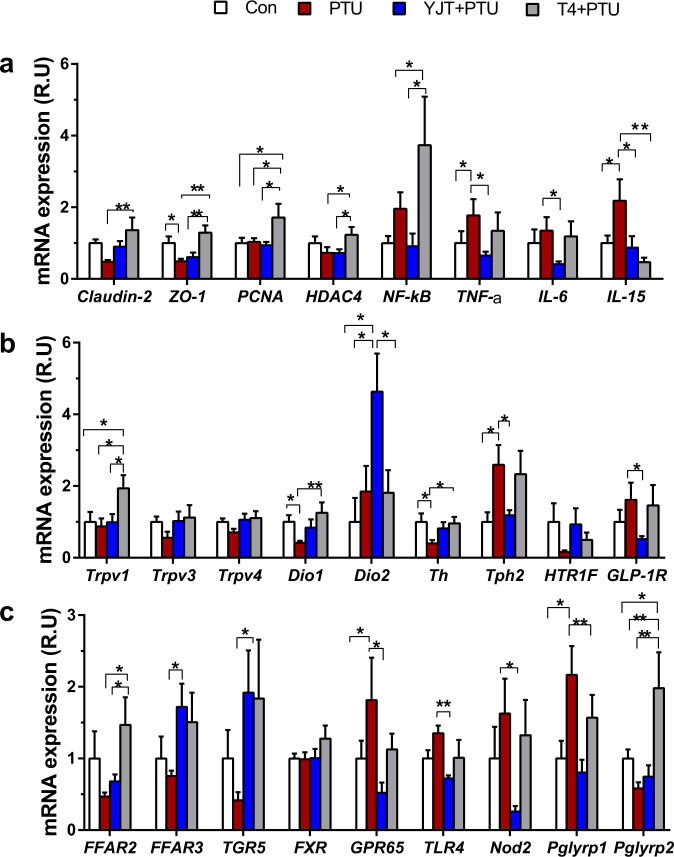
Changes in the relative mRNA expression of intestinal pro-inflammatory and inflammatory markers and metabolite-related receptors in response to different treatments. **a** The genes for intestinal tight junction markers *claudin-2* and zonula occludens-1 (*ZO-1*), proliferating cell nuclear antigen (*PCNA*), histone deacetylase 4 (*HDAC4*), and the inflammatory markers nuclear factor kappa-light-chain-enhancer of activated B cells (*NF-κB*), tumor necrosis factor-α (*TNF-α*), and interleukin 6 and 15 (*IL-6* and *IL-15*). **b** Transient receptor potential channel of vanilloid types 1, 3, and 4 (*Trpv1*, *Trpv3*, and *Trpv4*), type 1 and 2 iodothyronine deiodinase (*Dio1* and *Dio2*), tyrosine hydroxylase (*Th*), tryptophan hydroxylase 2 (*Tph2*), 5-hydroxytryptamine receptor 1 F (*HTR1F*) and glucagon-like peptide-1 receptor (*GLP-1R*). **c** Free fatty acid receptors 2 and 3 (*FFAR2* and *FFAR3*), G-protein-coupled bile acid receptor 5 (*TGR5*), farnesoid X receptor (*FXR*), G protein-coupled receptor 65 (*GPR65*), Toll-like receptor 4 (*TLR4*), Nod-like receptors 2 (*Nod2*), peptidoglycan recognition proteins 1 and 2 (*Pglyrp1* and *Pglyrp2*). The data are presented as means ± SEM (*n* = 7–8 per group). One-way ANOVA followed by post hoc LSD test. *P < 0.05, and **P < 0.01. Different letters above the columns indicate significant differences. Con, control group that received only saline; PTU, the rats that received 10 mg/kg propylthiouracil (PTU); YJT + PTU, the rats that received 2.1 g/kg YJT and 10 mg/kg PTU; T4 + PTU, the rats that were treated with 0.5 mg/kg L-thyroxine and 10 mg/kg PTU.

For further detection of the genes relevant for maintaining the intestinal barrier, gut signaling, and inflammatory cytokines that were modulated by YJT, the mRNA expressions of several key markers were quantified by a real-time quantitative polymerase chain reaction (RT-qPCR) in the ileum of the small intestine. Consistent with the histology of the ileum, the expression of claudin-2 and zonula occludens-1 (*ZO-1*, the epithelial tight junction molecules) tended to decrease or decreased significantly in PTU-treated rats compared to the controls. Additionally, these changes were partly or completely recovered by YJT and T4 treatments (Fig. 2a). In contrast with YJT, T4 treatment increased the expression of proliferating cell nuclear antigen (*PCNA*, a marker for cell proliferation, Fig. 2a) and histone deacetylase 4 (*HDAC4*, Fig. 2a). YJT treatment attenuated PTU-induced increases in the gene expression of pro-inflammatory and inflammatory cytokines including nuclear factor kappa-light-chain-enhancer of activated B cells (*NF-κB*), *TNF-α*, interleukin 6 (*IL-6*), and *IL-15*, whereas T4 treatment did not demonstrate this preventive effect on intestinal inflammation (Fig. 2a). Moreover, T4 treatment induced *NF-κB* expression (Fig. 2a), and stimulated the expression of transient receptor potential channel of vanilloid type 1 (*Trpv1*) rather than *Trpv3* or *Trpv4* (sensation of heat, inflammation and pain, Fig. 2b). Additionally, both YJT and T4 treatments reversed PTU-induced reduction in *Dio1* expression, and YJT even induced a 4.5-fold increase in *Dio2* expression (Fig. 2b), enhancing the conversion from inactive T4 to biologically active T3. The expression of tyrosine hydroxylase (*Th*, a rate-limiting enzyme for norepinephrine (NE) synthesis) decreased, and tryptophan hydroxylase 2 (*Tph2*, a rate-limiting enzyme for 5-hydroxytryptamine (5-HT) synthesis) increased in PTU-treated rats, which were reversed with YJT and T4 treatments (Fig. 2b). However, no difference was observed in the expression of 5-HT receptor *HTR1F* (Fig. 2b). In comparison with PTU-treated rats, YJT treatment inhibited *GLP-1R* expression together with a drop in the serum GLP-1 levels (Fig. 2b), relieving hypophagia in hypothyroidism. Further, YJT treatment increased the expression of free fatty acid receptors 3 (*FFAR3*) but not *FFAR2* (both are receptors of bacterial metabolites short-chain fatty acids, SCFAs), and increased Takeda G-protein-coupled receptor 5 (*TGR5*) but not farnesoid X receptor (*FXR*) (the latter two are receptors of bile acids, BAs) compared to that in the PTU-treated group (Fig. 2c). Additionally, significant down-regulations of intestinal sensors such as a pH-sensing G-protein-coupled receptor (*GPR65*), toll-like receptor 4 (*TLR-4*, detecting bacterial LPS), nucleotide-binding oligomerization domain 2 (*Nod2*, binding bacterial peptidoglycan), and peptidoglycan recognition protein 1 (*Pglyrp1*) were observed in the YJT-treated group compared with PTU-treated rats, whereas the up-regulation of *Pglyrp2* expression in the T4-treated group compared to all other groups is notable (Fig. 2c). These data indicate that YJT promotes intestinal gene expression extensively related with the enteroendocrine function, thyroid hormone conversion and multiple signaling pathways, and inhibits hypothyroidism-associated intestinal inflammation.

### YJT reverses hypothyroidism-associated intestinal microbiota dysbiosis

**Fig. 3:**
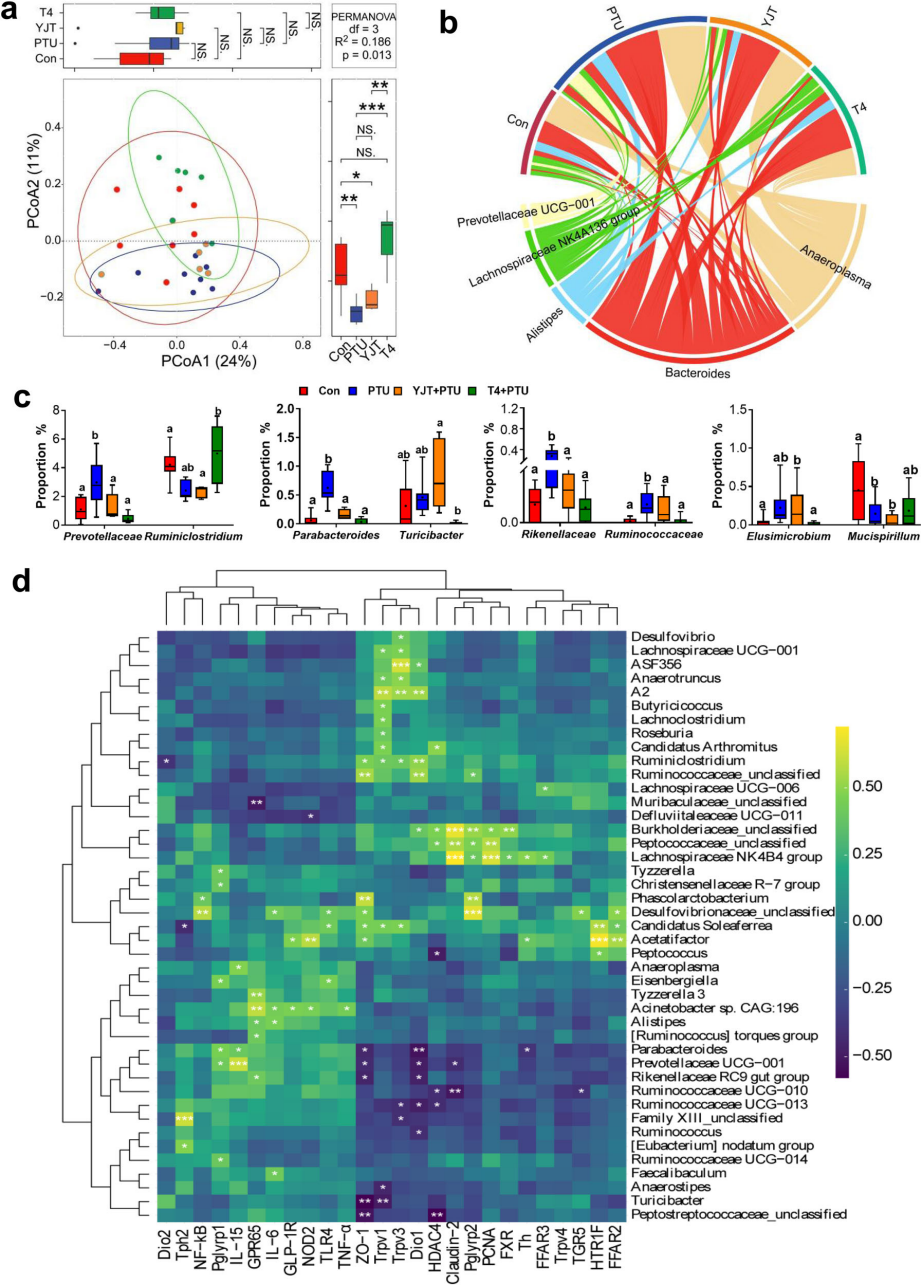
Fecal microbiota profile and their correlation with intestinal biomarkers. **a** The β diversity indicated by principal coordinate analyses (PCoA) based on the Bray–Curtis dissimilarity in amplicon sequence variant (ASV) abundances to analyze and visualize similarities and differences among samples. The boxplots on the top and right display the indices of Bray–Curtis dissimilarity along the PCo1 and PCo2 axis for subjects in each group, and statistical differences were determined by permutational multivariate analysis of variance (PERMANOVA). **b** The circleplot displaying the top 10 genera except for uncultured taxa. **c** The relative abundance of different bacteria at the genus level (the centre line represents the median of data, bounds of box represents the range of the middle 50% of data, and whiskers represent the spread of data beyond the box). **d** Heatmap of correlation coefficients between specific genera and mRNA expression of different intestinal markers and receptors. *P < 0.05, **P < 0.01, ***P < 0.001, and NS, non-significant. Different letters above the columns indicate significant differences. Con, control group that received only saline; PTU, the rats that received 10 mg/kg propylthiouracil (PTU); YJT + PTU, the rats that received 2.1 g/kg YJT and 10 mg/kg PTU; T4 + PTU, the rats that were treated with 0.5 mg/kg L-thyroxine and 10 mg/kg PTU.

To determine whether hypothyroidism disturbs the gut microbiota community and whether this disturbance could be prevented by YJT treatment, we analyzed 16 S rRNA gene sequences from the 26 fecal samples among the four experimental groups. The rarefaction curve of the Goods coverage index for the samples reached saturation (Supplementary Figure 2a), reflecting that numerous bacteria were all identified in the samples. Although the species diversity within the samples (α diversity) did not indicate differences between the treatments (Supplementary Table 1), the β diversity demonstrated segregation for the microbial community structure, as indicated by principal coordinate analyses (PCoA) based on the Bray–Curtis dissimilarity index (Fig. 3a). Each group exhibited specific core amplicon sequence variants (ASVs) across 85% of the samples in each group (Supplementary Figure 2b). The circleplot displayed the top 10 genera except for uncultured taxa (Fig. 3b). The biomarkers for different groups were identified through a linear discriminant analysis (LDA) coupled with the LDA effect size (LEfSe) (Supplementary Figure 2c). PTU-treated rats were affected by a higher proportion of genera such as *Prevotellaceae*, *Parabacteroides*, *Rikenellaceae*, and *Ruminococcaceae*, but exhibited a lower proportion of *Ruminiclostridium* and *Mucispirillum*, and these changes recovered to the control level with YJT or T4 treatments.

Next, we performed Pearson correlation and co-occurrence network analyses to reveal the potential correlations among biomarkers and between the gut microbiota and host biomarkers. Some genera such as *Parabacteroides*, *Prevotellaceae*, *Rikenellaceae*, *Ruminococcaeae*, *Lactobacillus,* and *Elusimiccobium* were observed to be negatively correlated, while *Ruminiclostridium* and *Ruminococcaceae unclassified*, and *Roseburia* were observed to be positively correlated with food intake, serum T4 levels and T_b_ (Supplementary Figure 2d and S2e). The intestinal sensors-*GPR65*, *TLR4*, *NOD2*, *Pglyrp1,* and *Pglyrp2* were positively correlated with each other and matched with inflammatory *IL-6* and *TNF-α* (Fig. 3e, S2f). Additionally, the intestinal epithelial tight junction markers (*claudin-2* and *ZO-1*) were negatively correlated with *Turicibacter*, *Ruminococcaceae UCG-010,* and *Peptococcaceae_unclassified*; additionally, *Turicibacter* was also negatively correlated with *Trpv1* (Fig. 3e, Supplementary Figure 3). Within this co-occurence network, the differential bacterial genera mainly generated three covarying enriched units, which are the genera: *Parabacterioides* belonging to f_*Tannerellaceae* (p_*Bacteroidota*), seven genera (in red font) belonging to f_*Ruminococcaceae* (p_*Firmicutes*), and nine genera (in yellow font) belonging to f_*Lachnospiraceae* (p_*Firmicutes*). These genera from the same p_*Firmicutes* are usually positively correlated with each other, whereas they are negatively correlated with the genus from p_*Bacteroidota*. Altogether, this broad co-expression network between differential bacteria genera and host phenotypes imply the potential role of gut microbes in regulating host feeding, *T*_b_, and intestinal barrier, and inflammation.

### CMT regains thermal and inflammatory phenotypes of the donors

**Fig. 4:**
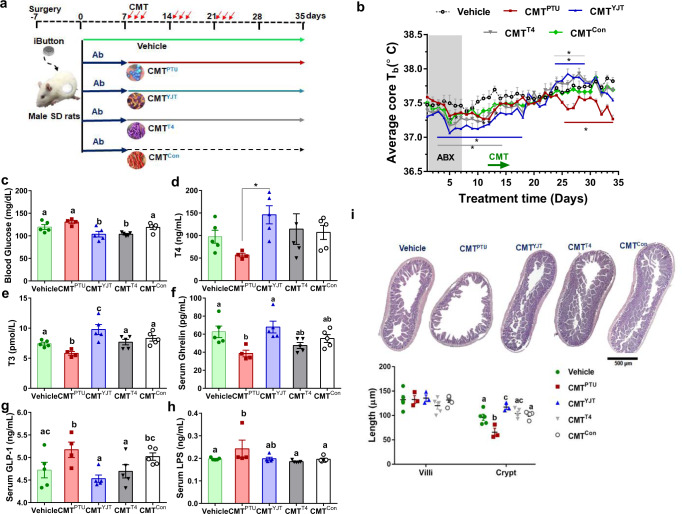
Cecal microbiota transfer (CMT) modulates the metabolic phenotypes, serum metabolites, and ileum histology in recipients. **a** Schematic overview of the experimental design. **b** Average core body temperature (*T*_b_) during the experiment process (*n* = 4 per group). **c** Blood glucose levels. **d**–**h** The serum thyroxine (T4), tri-iodothyronine (T3), ghrelin, glucagon-like peptide-1 (GLP-1), and lipopolysaccharides (LPS) by ELISA. **i** Histology assay for the ileum by hematoxylin and eosin staining for four experimental groups. The scale bar is 500 µm. The data are presented as means ± SEM. One-way ANOVA followed by post hoc LSD test. *P < 0.05 vs vehicle. Different letters above the columns indicate significant differences. Vehicle, the control rats which were orally gavaged with phosphate-buffered saline (PBS); CMT^PTU^, the rats that were colonized by cecal microbiota from PTU-treated donors; CMT^YJT^, the rats that were colonized by cecal microbiota from YJT + PTU-treated donors; CMT^T4^, the rats that were colonized by cecal microbiota from L-thyroxine+PTU -treated donors; CMT^Con^, the rats that were colonized with cecal microbiota from control donors.

To further confirm the roles of gut microbiota in mediating YJT-induced thermoregulation and inflammation-preventing hypothesis, we performed a CMT. The cecal microbiota from the PTU-treated, YJT + PTU-treated, T4 + PTU-treated, or Control rats were transferred to antibiotic-treated recipients, which were named as CMT^PTU^, CMT^YJT^, CMT^T4^, and CMT^Con^, respectively (Fig. 4a). As we predicted, the recipients exhibited similar thermal and inflammatory phenotypes to their donors (Figs. 4b, c). An antibiotic treatment for a duration of one week resulted in a chronic decrease in the T_b_ levels even 2 weeks after termination, with no significant changes in the body mass or food intake (Fig. 4b, Supplementary Figure 4a, b). The CMT^PTU^ rats demonstrated lower T_b_ and food intake levels than the vehicle group, while the CMT^YJT^ and CMT^T4^ rats did not show significant difference or even had higher T_b_ than that in the vehicle group (Fig. 4b). Moreover, the CMT^PTU^ rats exhibited higher blood glucose, serum GLP-1, and LPS levels, yet demonstrated lower serum T3, T4, ghrelin levels and lower crypt lengths in the ileum than that in the other groups (Fig. 4c–i).

### YJT–microbiota activates BAT thermogenesis and attenuates intestinal inflammation

**Fig. 5:**
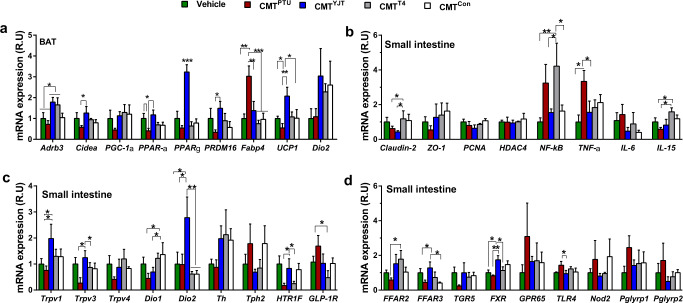
The expression of thermogenic markers in brown adipose tissue (BAT) and inflammatory regulators in the small intestine in CMT recipients. **a** Adrenergic receptor beta-3 (*Adrb3*), cell death-inducing DNA fragmentation factor-like effector A (*Cidea*), proliferator-activated receptor γ coactivator 1α (*PGC-1α*), proliferator-activate receptor alpha (*PPAR-α*), proliferator-activated receptor-gamma (*PPAR-γ*), positive regulatory domain containing 16 (*PRDM16*), fatty acid-binding protein 4 (*Fabp4*), uncoupling protein 1 (*UCP1*) and type 2 iodothyronine deiodinase (*Dio2*). **b** *Claudin-2*, zonula occludens-1 (*ZO-1*), proliferating cell nuclear antigen (*PCNA*), histone deacetylase 4 (*HDAC4*), nuclear factor kappa-light-chain-enhancer of activated B cells (*NF-κB*), tumor necrosis factor-α (*TNF-α*), and interleukin 6 and 15 (*IL-6* and *IL-15*). **c** Transient receptor potential channel of vanilloid types 1, 3, and 4 (*Trpv1*, *Trpv3*, and *Trpv4*), type 1 and 2 iodothyronine deiodinase (*Dio1* and *Dio2*), tyrosine hydroxylase (*Th*), tryptophan hydroxylase 2 (*Tph2*), 5-hydroxytryptamine receptor 1 F (*HTR1F*) and glucagon like peptide-1 receptor (*GLP-1R*). **d** Free fatty acid receptors 2 and 3 (*FFAR2* and *FFAR3*), G-protein-coupled bile acid receptor 5 (*TGR5*), farnesoid X receptor (*FXR*), G protein-coupled receptor 65 (*GPR65*), toll-like receptor 4 (*TLR4*), nod-like receptors 2 (*Nod2*), peptidoglycan recognition proteins 1 and 2 (*Pglyrp1* and *Pglyrp2*). One-way ANOVA followed by post hoc LSD test. *P < 0.05, **P < 0.01, and ***P < 0.001. The different letters above the columns indicate significant differences. Vehicle, the control rats which were orally gavaged with phosphate-buffered saline (PBS); CMT^PTU^, the rats that were colonized by cecal microbiota from PTU-treated donors; CMT^YJT^, the rats that were colonized by cecal microbiota from YJT + PTU-treated donors; CMT^T4^, the rats that were colonized by cecal microbiota from L-thyroxine+PTU-treated donors; CMT^Con^, the rats that were colonized with cecal microbiota from control donors.

The BAT is an important thermogenic organ with the unique mitochondrial protein uncoupling protein 1 (UCP1) in small mammals and also in humans^18,19^. We further quantified the mRNA expressions of thermogenesis-related genes in BAT, and genes with relevance in maintaining the intestinal barrier, intestinal hormones, nervous or immune signaling, and inflammation-related biomarkers in the ileum via RT-qPCR in the antibiotic-treated rats that were transferred with cecal microbiota from the PTU-treated, YJT + PTU-treated, T4 + PTU-treated, and Control rats. Regarding the changes in T_b_, the CMT^YJT^ group was observed to mostly show higher mRNA levels of *Adrb3* (beta 3-adrenergic receptors), *Cidea* (cell death-inducing DNA fragmentation factor-alpha (DFFA)-like effector a, which is a transcriptional coactivator), *PPAR-α* (proliferator-activate proliferator-activated receptor alpha), *PPAR-γ*, *PRDM16* (positive regulatory domain containing 16), and *UCP1* (uncoupling protein 1), and a lower level of *Fabp4* (fatty acid-binding protein 4) in the BAT than the CMT^PTU^ group, whereas other genes such as *PGC-1*α (proliferator-activated receptor γ coactivator 1α) and *Dio2* did not differ among groups (Fig. 5a). These data support that YJT-treated microbiota activated BAT thermogenesis function.

In the ileum, the expression of *claudin-2* was significantly higher in the CMT^T4^ group than in the CMT^PTU^ and CMT^YJT^ groups (Fig. 5b), accompanied by no differences in *ZO-1*, *PCNA,* or *HDAC4*. The CMT^YJT^ group had relatively lower *NF-κB* and *TNF-α* values than those in the CMT^PTU^ group, whereas both *NF-κB* and *IL-15* levels were higher in CMT^T4^ versus CMT^YJT^ rats (Fig. 5b). The expression of *Trpv1* and *Trpv3* was higher in the CMT^YJT^ group than in the CMT^YJT^ group, while *Trpv4* expression did not differ among groups (Fig. 5c). The expression of *Dio1* was lower both in the CMT^PTU^ and CMT^YJT^ groups than that in the CMT^Con^ group, whereas *Dio2* was higher in the CMT^YJT^ group when compared to that in the other groups (Fig. 5c). Both *Th* and *Tph* expression showed no differences in the groups; however, *HTR1F* was observed to express weakly in the CMT^PTU^ and CMT^T4^ groups (Fig. 5c). The *GLP-1R* expression was lower in the CMT^T4^ group than in the CMT^PTU^ group (Fig. 5c). Additionally, up-regulations in mRNA expression of the receptors, such as *FFAR2*, *FFAR3*, and *FXR* except *TGR5*, and a marked down-regulation in *TLR-4* were observed in the CMT^YJT^ group compared with the CMT^PTU^ group; *GPR65*, *Nod2*, *Pglyrp1* and *Pglyrp2* showed lower quantity in CMT^YJT^ vs. CMT^PTU^ rats (Fig. 5d). These results indicate that YJT-treated microbiota regulate bacteria-related multiple signaling pathways and attenuate hypothyroid-associated intestinal inflammation.

### Bacterial colonization confers regulation of thermogenesis and inflammation

**Fig. 6:**
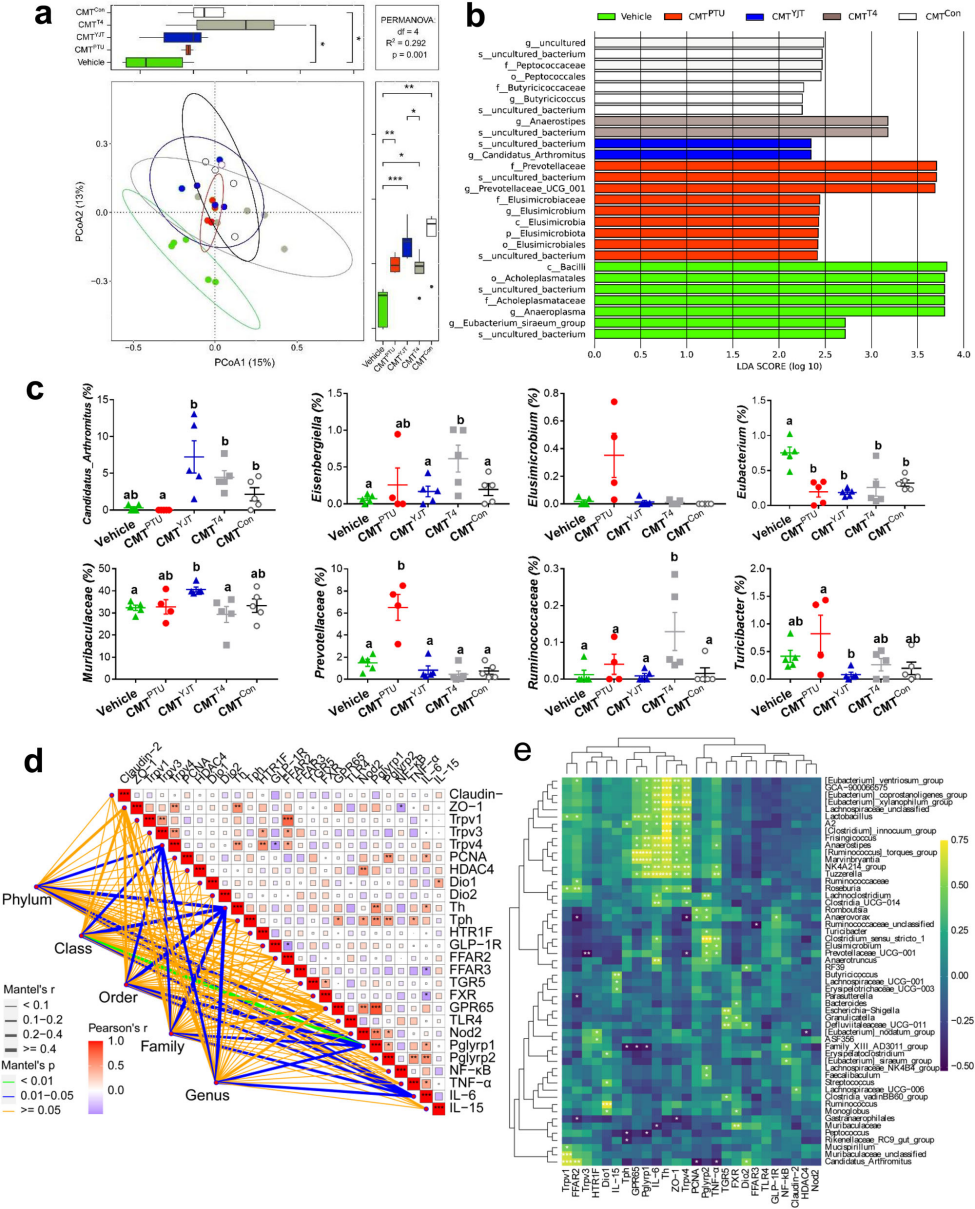
Cecal microbiota profile and their correlation with host biomarkers in CMT. **a** The β diversity indicated by principal coordinate analyses (PCoA) of Principal coordinate analyses (PCoA) based on Bray–Curtis dissimilarity in amplicon sequence variant (ASV) abundances to analyze and visualize similarities and differences among samples. The boxplots on the top and right display the indices of Bray–Curtis dissimilarity along the PCo1 and PCo2 axis for subjects in each group, and statistical differences were determined by permutational multivariate analysis of variance (PERMANOVA). **b** Differential bacterial taxonomy selected by LEfSe analysis with an LDA score >2 in the fecal microbiota community. **c** Relative abundance of different bacteria at the genus level (“+” indicates the mean of data). **d** Combinative plot of Mantel test and Pearson correlation between intestinal markers and bacteria taxonomy. **e** Heatmap of correlation coefficients between specific genera and mRNA expression of different intestinal markers and receptors. One-way ANOVA followed by post hoc LSD test. *P < 0.05, **P < 0.01, and ***P < 0.001. The different letters above the columns indicate significant differences. Vehicle, the control rats which were orally gavaged with phosphate-buffered saline (PBS); CMT^PTU^, the rats that were colonized by cecal microbiota from PTU-treated donors; CMT^YJT^, the rats that were colonized by cecal microbiota from YJT+ PTU-treated donors; CMT^T4^, the rats that were colonized by cecal microbiota from L-thyroxine+PTU-treated donors; CMT^Con^, the rats that were colonized with cecal microbiota from control donors.

We performed 16 S rRNA gene sequencing and analysis on the fecal samples of all donors and recipients to confirm efficiency of bacterial colonization and compare group differences in the recipients. Although α and β diversities of gut microbiota community in the recipients were different from those of the donors (Supplementary Figure 5a, b; Supplementary Tables 2–5), some specific genera, such as *Mucispirillum*, *Prevotellaceae*, and *Turicibacter*, exhibited similar patterns with those of the recipients (Supplementary Figure 5c). Moreover, the β diversity displayed separation between groups in the recipient rats (Fig. 6a). The bacterial biomarkers in each group were screened out in the recipients (Fig. 6b), and the CMT^YJT^ rats showed a higher relative abundance of *Candidatus_Arthromitus* and a lower abundance of *Prevotellaceae* and *Turicibacter* compared with CMT^PTU^ rats; whereas *Ruminococcaceae* was overexpressed in CMT^T4^ rats (Fig. 6c), indicating the diverse structures and compositions of gut microbiota community due to CMT from different donors (Fig. 6c).

Further, Pearson correlation and co-occurrence network analyses revealed potential correlations for the abundance of these gut bacteria and differential metabolic phenotypes such as serum T4 levels (Supplementary Figure 6a, b) and gene expression related to BAT thermogenesis (Supplementary Figure 6c, d) and intestinal inflammatory makers (Figs. 6d, e). The core T_b_ may be sensed by intestinal Trpv1 and regulated by serum T3 levels and BAT regulators (Supplementary Figure 7). The genus *Alistipes* was negatively correlated with intestinal *FFAR2* expression; additionally, *Lactobacillus* and *Eisenbergiella* were negatively correlated with serum GLP-1 and LPS levels, respectively (Supplementary Figure 7). Overall, co-occurrence network analyses reveal that the interaction of CMT-induced bacteria contributes to regulations of BAT thermogenesis and systemic inflammation.

## Discussion

Recently, despite extensive studies on HM as a promising agent to treat metabolism-related diseases, knowledge on the combination of HM and gut microbiome is limited, and several questions in this field that can help to maintain the human health status remain unanswered^15^. By combining these two interdependent fields in this study, we demonstrated that the administration of YJT exerted remarkable effects on T_b_ and thyroid hormone metabolism in rats with PTU-induced hypothyroidism. Meanwhile, based on the results of the 16 S rRNA gene sequencing and microbiota transfer, the effects of YJT could be interpreted and confirmed by shifts in the gut microbial community, and therefore, by the regulation of a specific set of receptors (e.g., FFAR2/FFAR3, TGR5/FXR, Pglyrp1/2/Nod2 and TLR4) that can be recognized as bacterial metabolites and cell wall components. We highlighted the YJT–microbiota–gut signaling cascade pathway in mediating the warm properties of YJT for activating thermogenesis and preventing systemic inflammation.

Hypothyroidism is one of the most common metabolic disorders associated with intestinal and systemic dysbiosis^20^. Rats with PTU-induced hypothyroidism reportedly have thyroid hormone levels similar to those of humans with hypothyroidism^6^. Therefore, in our study, hypothyroidism was induced by PTU treatment in Sprague–Dawley (SD) rats. As expected, PTU treatment led to a loss of appetite, inhibition of basal energy metabolism, and reduction in T_b_. These metabolic phenotypes were associated with increased circulating levels of anorexic intestinal hormone GLP-1, and low levels of orexigenic ghrelin, and reduced thyroid hormone levels in hypothyroid rats. Disorders in the gut microbiota community and enhancement of systemic inflammation were the most notable results in our PTU rat model. Moreover, the recipients that were transferred with microbiota from the hypothyroid rats demonstrated similar metabolic and inflammatory phenotypes as their donors, which was also observed in former studies^21^. Therefore, these data indicate the pathogenic role of hypothyroidism microbiota in shaping hypometabolism and hyperinflammation.

For interpreting these changes by considering the contribution of gut microbiota to the production of intestinal hormones and inflammatory cytokines, we detected the mRNA expression of some of the most important receptors and biomarkers in the small intestine. Notably, the innate pattern recognition receptors, such as Nod2, Pglyrp1, 2 (peptidoglycan sensors), and TLR4 (LPS receptor), are essential for mammalian inflammatory responses and the production of antimicrobial peptides^22^. We observed significant increases in the expressions of *GPR65*, *Nod2*, *Pglyrp1*, and *TLR4* in PTU-treated rats. These receptors, via recognizing bacterial components (peptidoglycan and LPS), activated inflammatory responses, breached the intestinal barrier, and reduced appetite by increasing GLP-1 secretion^23–25^. Moreover, bacterial LPS has been demonstrated to inhibit the Dio activity and decrease the level of T3 in the circulation, and thus, lead to hypothermia^26–28^. Additionally, the present data support that *Roseburia*, *Lachnospiraceae*, *Peptococcaceae*, and *Ruminiclostridium* were positively related with T_b_, whereas *Parabacteroides*, *Rikenellaceae*, and *Ruminococcaceae* were negatively correlated with T_b_. Consistent with these data, we previously detected similar changes in the flora of the gerbil model of hypothyroidism^29^. Further, decreases in the length of villi, crypt depth, and intestinal tight junction molecules in hypothyroid rats demonstrated a disturbance in intestinal absorption and barrier leading to increased systemic inflammatory responses^30^. Altogether, intestinal microbiota dysbiosis through the activation of LPS–TLR4 and peptidoglycan–Nod2/Pglyrp1 signaling pathways may be related to reduced metabolism and enhanced systemic inflammation in hypothyroid rats.

Treatment with L-thyroxine as a conventional method for the therapy of hypothyroidism remedied the loss of body mass and food intake in hypothyroid rats. Meanwhile, based on the recommended dosage in former studies, the current data indicate remarkable increases in the food intake and T_b_ levels in T4-treated rats; increased weights of some organs, including the spleen, heart, and liver; enhanced length of villi and crypt and increased intestinal cell proliferation; and the overexpression of several pro-inflammatory and inflammatory markers in these T4-treated rats. HDAC4, one of the enzymes involved in post-translational modification, was reported to stimulate cell proliferation and enhance the epithelial tight junction^31^, supporting our finding of high expression in intestinal *HDAC4*, *PCNA,* and tight junction markers in T4-treated rats. These data indicate that T4 treatment as therapy for hypothyroidism generates adverse events, such as hyperphagia, hyperthermia, and hyperinflammation.

In this study, the administration of YJT in drug-induced hypothyroid rats resolved the reduction of T_b_ and prevented systemic inflammation. While the exact composition and active components of YJT are not fully understood, there is some evidence to suggest that it contains compounds with antioxidant and anti-inflammatory properties, such as flavonoids and liquiritigenin, as well as prebiotic polysaccharides^16^. These bioactive components may be directly sensed by gut epithelial TRP channels, regulate the secretion of hormones (such as GLP-1, leptin, and adiponectin) and contribute to their potential benefits on BAT and related pathways^32,33^. Alternatively, these plant-derived products may be fermented into SCFAs to exert anti-inflammatory and thermogenic effects in the body^34^. To investigate whether the gut microbiota played a role in the beneficial effects of YJT administration on hypothyroid symptoms, we transferred the cecum microbiota from the YJT-treated donors to the antibiotic-treated rats. Antibiotic treatment could not deplete microbiota completely, but reduced the existing microbial community for the purpose of removing influence of the recipients’ inherent microbiota, and created a receptive environment for colonization by donors’ microbiota, which has been applied effectively for investigation of the function played by the gut microbiota^35,36^. Although the donors’ microbiota could not colonize totally in the recipients’ intestines because of competitions with inherent microorganisms, some specific genera exhibited similar changing patterns with the donors. The same procedure of CMT was also applied in the previous studies^37^. These data demonstrate the engraftment efficiency and that the recipients harvest similar metabolic and inflammatory phenotypes with the donors.

The transfer of microbiota may also deliver the efficacy of YJT on the expression of inflammation-related genes in the small intestine and thermogenesis-related genes in BAT. The increased gene expression of the rate-limiting enzyme (*Th*) for NE synthesis in the CMT^YJT^ rats contributes to the activation of BAT thermogenesis. In addition, the increasing expression of intestinal Dio2, which promotes conversion from inactive T4 to the biologically active T3^1,29^, was also considered to induce thermogenesis in CMT^YJT^ rats. These findings suggest that YJT or YJT-treated microbiota may affect the production and processing of these neurotransmitters and hormones in the body, which in turn may contribute to its warm properties. Our data further indicate that bacterial metabolite-related receptors, such as the FXR and/or TGR5 (for BAs) and the FFAR2 and/or FFAR3 (for SCFAs) may be involved in the regulation of intestinal neurotransmitters and hormones. Previous studies have suggested the importance of BA-TGR5/FXR-Dio signaling in enhancing energy expenditure and glucose control^38–40^. Additionally, our data support that the reduction in intestinal LPS–TLR4 and peptidoglycan–Nod2/Pglyrp1 signaling may convey the anti-inflammatory effect of YJT or YJT-treated microbiota. Furthermore, an increase in the *Trpv1* and *Trpv3* expression in the recipients of YJT-treated microbiota is involved in the sensation of inflammation, pain, and heat and consequent regulations of T_b_^41,42^. Considering the previous literature and current data, *Oscillibacter* which demonstrated a positive correlation with Dio1 expression may enhance the anti-inflammatory response^43^. Additionally, the decrease in the *Firmicutes* to *Bacteroidetes* ratio is another change that we observed in the YJT-treated group, which was previously associated with a putative disease state^44^. Our study demonstrates that YJT-treated gut microbiota can exert effects on the activity of the intestinal innate immune system and protect animals against hypothyroidism-induced hyperinflammation. Moreover, the data suggest a critical role of the microbiota–gut–BAT axis in mediating YJT-induced thermoregulation.

**Fig. 7:**
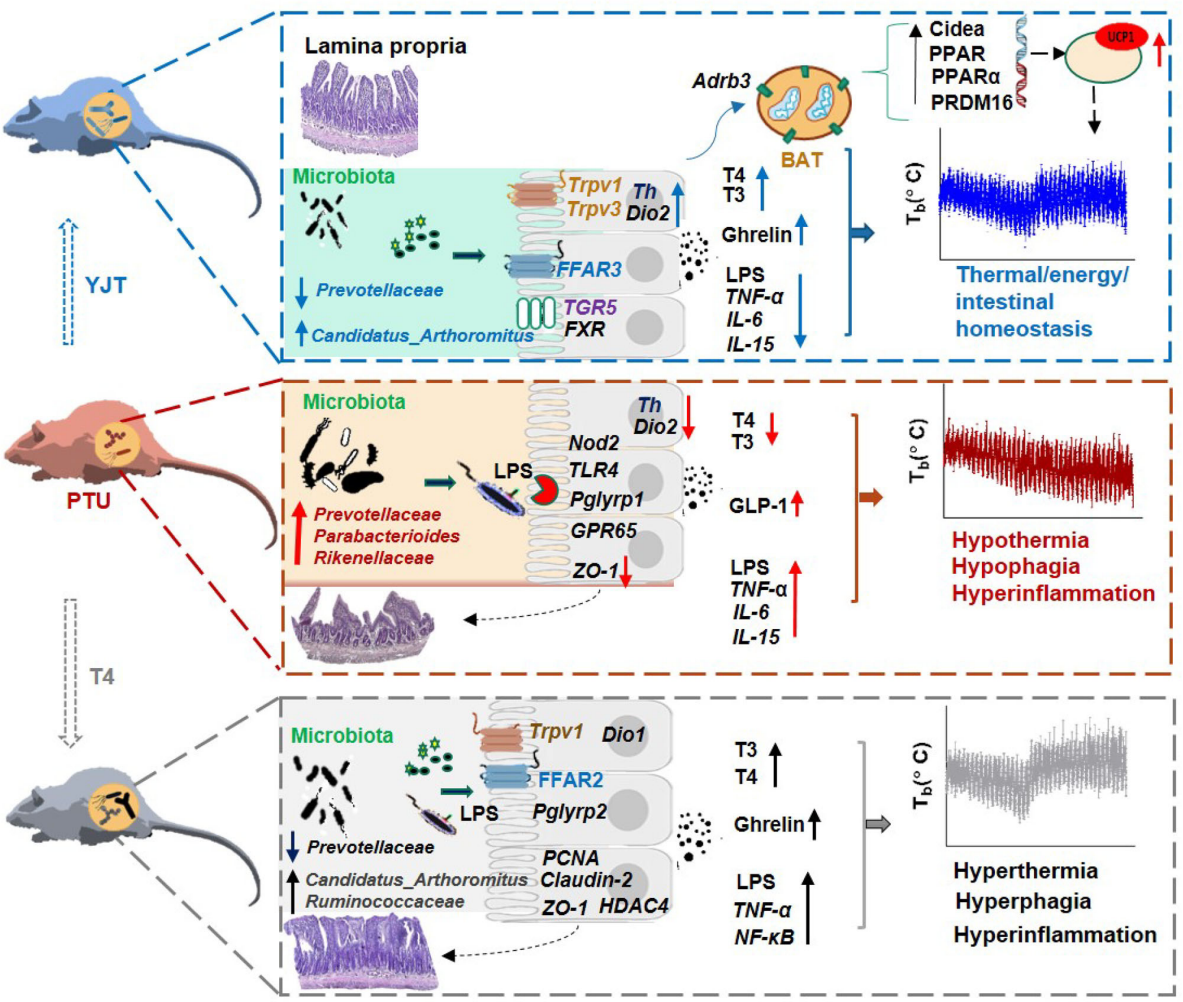
The paradigm summarizing the thermogenic and anti-inflammatory properties of YJT via the gut microbiota and related multiple signaling pathways. Propylthiouracil (PTU)-treated hypothyroid rats exhibit hypothermia, hypophagia, and hyperinflammation, which have been associated with intestinal microbiota dysbiosis, particularly with extremely enriched *Prevotellaceae*. As a prebiotic, *Yijung-tang* (YJT) modulates the gut microbiota and metabolite-related receptors (e.g., FFAR3 and TGR5/FXR) to stimulate gene expression of intestinal tyrosine hydroxylase (*Th*) for norepinephrine synthesis and iodothyronine deiodinases 2 (*Dio2*) for thyroid hormone metabolism, and consequently activates the thermogenic pathway in brown adipose tissue (BAT) and attenuates hypothyroidism-related systemic inflammation. In contrast, L-thyroxine (T4) treatment in hypothyroid rats stimulates ghrelin secretion and activates intestinal LPS–TLR4 and peptidoglycan–Pglyrp2 signaling pathways, and leads to hyperthermia, hyperphagia, and hyperinflammation.

Thus, YJT may act as a prebiotic by shifting the gut microbiota taxa, elevate the T_b_ and alleviate hypothyroidism-related inflammatory responses. These effects on thermogenesis may be related to the increased expression of *Th* and *Dio2* which are involved in the synthesis of norepinephrine and the metabolism of thyroid hormones in the small intestine, ultimately activating the thermogenic pathway in the BAT. In contrast to the conventional drug L-thyroxine for curing hypothyroidism, YJT has a unique efficacy for attenuating systematic inflammatory responses via the suppression of intestinal LPS–TLR4 and peptidoglycan–Nod2/Pglyrp1 signaling pathways (Fig. 7). Altogether, the current reported data suggest that the beneficial thermogenic and anti-inflammatory properties of YJT may be associated with alteration in the gut microbiota, via the microbiota–gut–BAT axis. Caution should be taken in interpreting these findings due to the limited number of individuals per group. The exact active components of YJT and the mechanisms by which to regulate thermal, energy, and intestinal homeostasis should still be fully characterized for understanding its prebiotic function. Given the widespread nature of HM in human health, these findings can open a new window to the modern medicinal system by strengthening the rationale for a paradigm shift to empower holobiont-centric medicine.

## Methods

### Extraction and preparation of YJT

YJT was procured from the medical supply store of Dongguk University International Hospital (Ilsan, Goyang-si, Republic of Korea). The dried plant materials were ground into an appropriate coarse powder using a home grinder, immersed in a 10-fold volume of 30% ethanol (v/v), and boiled for 2 h. The solution was subsequently cooled at room temperature for 1 h and subjected to centrifugation at 1700 × *g* for 20 min to collect the supernatant. The supernatant was filtered through a Whatman filter and then evaporated under reduced pressure at 50 °C using a rotary evaporator (EYELA N-1200A, EYELA, Tokyo, Japan) to obtain the initial extract. This initial aqueous product was freeze-dried using a lyophilizer (Bondiro, IlshinBioBase, Dongducheon, Republic of Korea), and the final extract was stored at −80 °C until further use. The doses of YJT were calculated as an equivalent dose ratio of human to rat according to the previous instructions^45^. Therefore, the equivalent single doses of YJT for rats were calculated as 2.1 g/kg/day.

### Experimental animals

Three-week-old male Sprague-Dawley (SD) rats (with body mass of ~100 g), were purchased from DBL (277 Deokho-ro, Eumseong-jun, Chung Cheong buk-do, Republic of Korea). After 7 days of quarantine to prevent microbial transmission and monitor the food intake accurately, all the animals were separated into individual cages and provided standard rat pellet chow (Carjill Ajri Purina, Seongnumsi, Gyeonggi-do, Republic of Korea) and water *ad libitum*. They were housed under standard laboratory conditions at an ambient temperature of 22 ± 3 °C with a humidity of 60 ± 5% and a daily 12/12 h light/dark cycle during the course of the experiment. All the procedures in the study were approved by the Institutional Animal Care and Use Committee of Dongguk University (approval number: IACUC-202201223) and were performed in compliance with the ‘Guide for the Care and Use of Laboratory Animals’.

### Experimental designs

Experiment 1 was designed to test the effects of YJT on T_b_, gene expression, serum metabolites, and the gut microbial community. In the first set of rats, the abdomens were implanted with Thermochron iButton, and at 1 week postoperatively, the animals were divided into four groups. A control group (Con) was subcutaneously injected with 0.3 mL saline/animal during the experimental period, and the other 3 groups of rats were administrated a daily subcutaneous injection of 10 mg PTU/kg body mass into the dorsal neck for a period of 2 weeks (Treatment 1)^6,7^. These 3 groups were then under different treatments. The PTU-treated group only received PTU, and the other two groups received not only PTU but also 0.5 mg/kg/day L-thyroxin (T4, as a reference drug) or 2.1 g/kg/ day YJT for 4 weeks (Treatment 2) (Fig. 1a). The body mass (±0.1 g) and food consumption were monitored during the course of the experiment. At the end of the experimental period, fresh feces were collected from each rat, frozen in liquid nitrogen, and stored at −80 °C for DNA extraction. Blood samples were collected from the infraorbital vein and then centrifuged at 1500 × *g* for 30 min to obtain serum. After treatment, the rats were fasted for 12 h and were sacrificed under anesthesia by the administration of Zoletil® (tiletamine–zolazepam, Virbac, Carros, France) and Rompun® (xylazine–hydrochloride, Bayer, Leverkusen, Germany) according to a previous protocol^46^. Cecal contents were collected from each rat, frozen in liquid nitrogen, and stored at −80 °C for the experiment of CMT. The small intestine was excised, snap-frozen in liquid nitrogen, and stored at −80 °C for subsequent measurements.

Experiment 2 through CMT was designed to examine the role of gut microbiota in mediating the effects of YJT on T_b_, gene expression, and serum metabolites in PTU-induced hypothyroid rats. The other set of rats who received the iButton implantation was divided into 5 groups after a recovery period of 1 week (Fig. 4a). For sham CMT, the recipient rats received an intragastric gavage of 500 μL sterile phosphate-buffered saline (PBS, vehicle) during the experiment and were named as vehicle group. The other 4 groups were all administered an antibiotic cocktail (100 mg/kg streptomycin, 200 mg/kg ampicillin, 200 mg/kg neomycin, 200 mg/kg metronidazole, and 100 mg/kg vancomycin) via intragastric gavage once daily (500 μL per day) for 7 days^47^. These antibiotic-treated rats were then randomly transferred with the cecal microbiota from the PTU-, YJT + PTU-, and T4 + PTU-treated, and control donors via intragastric gavage (500 μL per day) for 3 days in a week (continuously for 3 weeks), and named as CMT^PTU^, CMT^YJT^, CMT^T4^, and CMT^Con^, respectively (Fig. 4a). Fresh feces were collected, frozen in liquid nitrogen, and stored at −80 °C for later DNA extraction. The small intestine and BAT were also immersed in liquid nitrogen and stored at −80 °C for further analysis.

### CMT

Cecal contents were collected from the PTU-, YJT + PTU-, and T4 + PTU-treated and control groups in Exp. 1, respectively. These cecal contents (200 mg) from 3 donors of each group were combined, diluted in 2 mL sterile PBS, homogenized for 15 s, and centrifuged (500 × *g*, 4 °C, 5 min) to remove large food residues^37,48,49^. Subsequently, a 500 μL suspension was delivered by intragastric gavage to the recipient rats (Exp. 2). For sham CMT (vehicle group), the rats received intragastric gavage of 500 μL sterile PBS to match the stress of gavage manipulation during the same days as CMT groups.

### Core T_b_

We used a non-invasive, continuous, longitudinal monitoring of core T_b_ in the physiological context^49,50^. In brief, Thermochron iButton, DS1922L-F5# (with a precision of 0.0625 °C) was programmed to start recording T_b_ 1 week after implantation at 30- or 60-minute intervals and then coated with a thin layer of paraffin wax for waterproofing (3:1 ratio). The iButton was implanted surgically into the peritoneal cavity under general anesthesia (by inhaling 3–5% isoflurane). After the surgery, each animal was returned to their home cage and allowed to recover for 1 week. At the end of the experiment, the logger was removed from the animal, and all the records were read using the OneWireViewer Software.

### Glucose tolerance test

Glucose tolerance was assessed by a general method described in former studies^51^. Briefly, on day 30 of the experiment, the rats were orally gavaged by 2 g/kg body mass of sterilized aqueous glucose solution in a state of fasting (12 h) (Sigma-Aldrich, St. Louis, MO, USA). Subsequently, the blood samples were collected from the rat’s tails by a small scratch, and the glucose levels were determined using a glucometer (Accu-Chek Active; Roche Diagnostics GmbH, Mannheim, Germany) at 0, 15, 30, 60, and 120 min after glucose administration.

### Serum biochemical parameter assays

We used enzyme-linked immunosorbent assay kits (ELISA) to estimate the levels in the serum, including TSH (CSB-E05115r), free Tri-iodothyronine (free-T3, CSB-E05076r), thyroxine (T4, CSB-E05082r), GLP-1 (CSB-E08117r), TNF-α (CSB-E11987r), ghrelin (CSB-E09816r), and LPS (CSB-E14247r) following the manufacturer’s recommendations (Cusabio, Wuhan, China). The detailed method for GLP-1 assays as an example was as follows^52,53^. The serum was first treated with inactivate dipeptidyl peptidase, which was pre-coated onto the surface of a microplate, and incubated for 2 h at 37 °C. A second, biotin-conjugated antibody specific for binding to GLP-1 was added to the microplate and incubation lasted for 1 h at 37 °C. Then, the avidin-conjugated horseradish peroxidase was added to the wells, and incubated for another 1 h at 37 °C. Following washes, a substrate solution was added to the wells, and color developed in proportion to the amount of GLP-1 binding in the initial step. The absorbance was measured by an ELISA reader (TECAN Spark, Greenmate Biotech Co, Switzerland) at 450 nm. The intra- and inter-assay CVs were <15% for free-T3 and T4 kits and in other kits, intra- and inter-assay CVs were <8% and <10%, respectively.

### RNA extraction and expression measurement via RT-qPCR

The total RNA was extracted from the BAT and small intestine using Trizol reagents (Bioline Reagent, London, UK) according to the manufacturer’s instructions. The total RNA was quantified by measuring the optical densities using a nanodrop spectrophotometer (Implen, Munich, Germany). cDNA was synthesized by reverse transcription of 1 μg of extracted RNA using an oligo-(dT) 18 primer (Thermo Fisher Scientific, Waltham, MA, USA) and RT PreMix kit (Bioneer, Daejeon, Korea). Real-time PCR was performed on a Light Cycler480TM device (Roche Applied Science, Basel, Switzerland) in a 96-well plate using a SYBR® Green real-time PCR Master Mix (Toyobo, Tokyo, Japan) and specific primer sets for different genes (Supplementary Table 6). The relative gene expression was calculated using the 2^−ΔΔCt^ method^54^, which was normalized against glyceraldehyde-3-phosphatase dehydrogenase (*GAPDH*).

### Histomorphology of the ileum

Fresh ileum tissues were fixed in a 4% paraformaldehyde solution. After being embedded in paraffin wax, the samples were serially sectioned at 4 μm using Leica RM2235 microtome (Leica Microsystems, Nussloch, Germany) and stained with hematoxylin–eosin. The sections were observed under the light microscope (BX61 Olympus, Tokyo, Japan) at 40× magnifications. Images of the ileum were captured using a digital camera (Olympus) and were displayed on a computer connected to the microscope. The histological parameters, such as the villus height and crypt depth in the tissue sections, were analyzed using the ImageJ software (Bethesda, MD, USA)^51,55,56^.

### 16 S rRNA gene amplicon sequencing and analysis

The total DNA was extracted from fecal pellets using QIAamp® fast DNA stool kit (Germany) according to the manufacturer’s protocols. The quality and quantity of DNA were detected using a nanodrop spectrophotometer (Implant, Munich, Germany) by measuring the A260/A280 ratio. Only DNAs with an A260/A280 ratio of 1.8–2.0 were used for PCR amplification. The V3–V4 hypervariable regions of the 16 S rRNA gene were amplified using two universal primers (341F–805 R) (Supplementary Table 7, 8)^37^. A PCR analysis was performed for each DNA sample in triplicate in the thermal cycler system (MiniAmp™ Thermal Cycler, Thermo Fisher). The PCR products were checked using electrophoresis in 1% (w/v) agarose gels in Tris, boric acid, ethylenediaminetetraacetic acid buffer stained with ethidium bromide and visualized under ultraviolet light. The PCR products were purified using a QIAamp® fast PCR purification kit (Germany) according to the manufacturer’s instructions and were pooled. Subsequently, sequencing was performed on an Illumina HiSeq 2500. The 16 S sequence paired-end data set was joined, and the quality was filtered using the FLASH method^57^. After the sequencing, we performed all sequencing analyses using QIIME2 according to the QIIME tutorial (http://qiime.org/) with some modified methods. The row sequences were jointed and selected (low-quality tags and chimeras that did not meet the length requirement were removed).

### Statistical analysis

The data of food intake and organ mass were analyzed by one-way analysis of covariance (ANCOVA) with body mass as a covariate. Serum hormones, *T*_b_, and other data of mRNA expression were analyzed by one-way analysis of variance (ANOVA). Significant group differences were further evaluated using post hoc analyses and least significant difference (LSD) tests when the main effects were significant and where required. The SPSS 17.0 software (SPSS Inc., Chicago, IL, USA) was used for all statistical analyses. The results are presented as mean ± SEM, and a *P* value < 0.05 was considered significant. GraphPad Prism 7.04 (GraphPad, San Diego, CA, USA) was used to create graphs.

Following the previous methods with appropriate modifications^48^, we assessed the richness and diversity of bacteria community (α diversity) for Chao 1, observed ASVs, Shannon index, and PD whole tree. The β diversity was estimated by PCoA based on the Bray–Curtis dissimilarity in ASV abundances, and statistical differences were determined by permutational multivariate analysis of variance (PERMANOVA). The specific bacteria were identified via STAMP^58^, and significant group differences in bacteria relative abundances were examined by one-way ANOVA followed by LSD tests where required. We used the LDA effect size coupled with LEfSe method to assess the differences in microbial communities using an LDA score threshold of 2. Venn diagrams were created using jvenn^59^.

### Reporting summary

Further information on research design is available in the Nature Research Reporting Summary linked to this article.

## Supplementary information

Supplementary Information

Reporting Summary

## Data Availability

The raw data of 16 S rRNA gene amplicon sequence are available in the NCBI Sequence Read Archive under accession PRJNA938178.

## Code availability

The codes that were used in the 16 S rRNA gene amplicon sequencing and analysis are available in the supplementary information file.
