# Supplementary material for: Yijung-tang improves thermogenesis and reduces inflammation associated with gut microbiota in hypothyroid rats
Source: NPJ Biofilms Microbiomes. 2023 Jun 3;9:32. doi: 10.1038/s41522-023-00396-2 (PMC10239510; doi:10.1038/s41522-023-00396-2)
Supplement: Supplementary file 1 — Supplementary Information [file 41522_2023_396_MOESM1_ESM.pdf]

Yijung-tang improves thermogenesis and reduces inflammation being associated with gut microbiota in hypothyroid rats

Saeid Khakisahneh, Xue-Ying Zhang, Song-Yi Han, Eun-Ji Song, Young-Do Nam, Hojun Kim

Supplementary Information

Supplementary Table 1. Alpha diversity indicated by Shannon index, PD whole tree, Chao1, and Observed ASVs in Exp. 1.

	Shannon index	PD whole tree	Chao1	Observed ASVs
Control	6.39±0.13	10.17±0.57	219.57±23.54	219.57±23.54
PTU	6.31±0.14	11.03±0.58	249.25±26.83	249.25±26.83
YJT+PTU	6.33±0.10	11.17±1.04	257.6±34.55	257.60±34.55
T4+PTU	6.48±0.09	11.19±0.42	267.67±14.91	267.67±14.91
F	0.364	0.58	0.657	0.657
P	0.78	0.635	0.587	0.587

Data are presented as means ± SEM. ASVs, amplicon sequence variants; PTU, the rats that received 10 mg/kg/body Propylthiouracil (PTU); YJT+PTU, the rats that received 2.1g/kg YJT and 10 mg/kg PTU; T4+PTU, the rats that were treated with 0.5 mg/kg L-Thyroxine and 10 mg/kg PTU.

Supplementary Table 2. Alpha diversity indicated by Shannon index, PD whole tree, Chao1, and Observed ASVs in the recipient rats in Exp. 2.

	Shannon index	PD whole tree	Chao1	Observed ASVs
Vehicle	6.14±0.21	9.94±0.82	219.73±26.00	217.20±25.45
CMT^{PTU}	6.01±0.16	9.00±0.52	183.66±15.02	180.50±14.38
CMT^{YJT}	6.24±0.12	9.96±0.59	220.10±27.11	219.00±26.61
CMT^{T4}	6.39±0.23	10.38±0.95	246.02±37.84	244.40±36.97
CMT^{Con}	5.98±0.15	8.67±0.53	184.08±16.76	182.80±16.34
F	0.500	0.431	0.439	0.408
P	0.871	1.002	0.985	1.050

Data are presented as means ± SEM. ASVs, amplicon sequence variants; Vehicle, the control rats which were orally gavaged with phosphate buffered saline; CMT^{PTU}, the rats that were colonized by cecal microbiota from PTU-treated donors; CMT^{YJT}, the rats that were colonized by cecal microbiota from YJT+PTU-treated donors; CMT^{T4}, the rats that were colonized with cecal microbiota from L-thyroxin+PTU-treated donors; CMT^{Con}, the rats that were colonized with cecal microbiota from control donors.

Supplementary Table 3 The relative abundence of the top 20 ASVs in the donors and recipient rats.

ASVs	D_Con	D_PTU	D_YJT	D_T4	R_Con	R_PTU	R_YJT	R_T4	Taxonomy
ASV_2	6%	9%	10%	6%	0%	4%	0%	0%	k__Bacteria; p__Firmicutes; c__Bacilli; o__Acholeplasmatales; f__Acholeplasmataceae; g__Anaeroplasma
ASV_1	4%	7%	10%	9%	9%	10%	10%	8%	k__Bacteria; p__Bacteroidota; c__Bacteroidia; o__Bacteroidales; f__Bacteroidaceae; g__Bacteroides; s__Bacteroides_vulgatus
ASV_8	1%	4%	1%	1%	1%	4%	0%	1%	k__Bacteria; p__Bacteroidota; c__Bacteroidia; o__Bacteroidales; f__Prevotellaceae; g__Prevotellaceae_UCG-001
ASV_3	3%	4%	3%	4%	6%	3%	6%	5%	k__Bacteria; p__Bacteroidota; c__Bacteroidia; o__Bacteroidales; f__Bacteroidaceae; g__Bacteroides
ASV_4	2%	3%	2%	3%	4%	5%	3%	4%	k__Bacteria; p__Bacteroidota; c__Bacteroidia; o__Bacteroidales; f__Rikenellaceae; g__Alistipes
ASV_5	3%	3%	4%	0%	0%	2%	4%	0%	k__Bacteria; p__Bacteroidota; c__Bacteroidia; o__Bacteroidales; f__Muribaculaceae; g__Muribaculaceae
ASV_9	3%	2%	2%	3%	0%	0%	1%	1%	k__Bacteria; p__Bacteroidota; c__Bacteroidia; o__Bacteroidales; f__Muribaculaceae; g__Muribaculaceae
ASV_6	2%	2%	2%	2%	3%	2%	3%	3%	k__Bacteria; p__Bacteroidota; c__Bacteroidia; o__Bacteroidales; f__Bacteroidaceae; g__Bacteroides
ASV_14	3%	2%	1%	1%	0%	1%	1%	0%	k__Bacteria; p__Firmicutes; c__Clostridia; o__Lachnospirales; f__Lachnospiraceae
ASV_12	2%	2%	2%	0%	0%	3%	4%	0%	k__Bacteria; p__Bacteroidota; c__Bacteroidia; o__Bacteroidales; f__Muribaculaceae; g__Muribaculaceae
ASV_10	1%	1%	1%	1%	3%	2%	2%	2%	k__Bacteria; p__Bacteroidota; c__Bacteroidia; o__Bacteroidales; f__Muribaculaceae; g__Muribaculaceae
ASV_18	2%	1%	2%	0%	0%	0%	1%	0%	k__Bacteria; p__Bacteroidota; c__Bacteroidia; o__Bacteroidales; f__Muribaculaceae; g__Muribaculaceae
ASV_13	1%	1%	1%	1%	2%	2%	2%	2%	k__Bacteria; p__Bacteroidota; c__Bacteroidia; o__Bacteroidales; f__Muribaculaceae; g__Muribaculaceae
ASV_15	1%	1%	1%	1%	3%	1%	2%	1%	k__Bacteria; p__Bacteroidota; c__Bacteroidia; o__Bacteroidales;

									f__Muribaculaceae; g__Muribaculaceae
ASV_17	1%	1%	1%	1%	2%	2%	3%	1%	k__Bacteria; p__Bacteroidota; c__Bacteroidia; o__Bacteroidales; f__Muribaculaceae; g__Muribaculaceae
ASV_41	1%	1%	0%	0%	2%	0%	0%	1%	k__Bacteria; p__Firmicutes; c__Clostridia; o__Lachnospirales; f__Lachnospiraceae; g__Lachnospiraceae_NK4A136_group
ASV_11	1%	1%	1%	1%	2%	1%	2%	3%	k__Bacteria; p__Bacteroidota; c__Bacteroidia; o__Bacteroidales; f__Bacteroidaceae; g__Bacteroides; s__Bacteroides_uniformis
ASV_7	2%	1%	3%	1%	2%	1%	4%	1%	k__Bacteria; p__Bacteroidota; c__Bacteroidia; o__Bacteroidales; f__Muribaculaceae; g__Muribaculaceae
ASV_37	0%	1%	1%	0%	0%	0%	0%	0%	k__Bacteria; p__Bacteroidota; c__Bacteroidia; o__Bacteroidales; f__Muribaculaceae; g__Muribaculaceae
ASV_22	1%	1%	1%	1%	1%	1%	1%	1%	k__Bacteria; p__Bacteroidota; c__Bacteroidia; o__Bacteroidales; f__Bacteroidaceae; g__Bacteroides; s__Bacteroides_acidifaciens

ASVs, amplicon sequence variants; D_Con, the donors of control group that just received phosphate buffered saline (PBS); D_PTU, the donors that received 10 mg/kg Propylthiouracil (PTU); D_YJT (D_YJT+PTU), the donors that received 2.1g/kg YJT and 10 mg/kg PTU; D_T4 (D_T4+PTU), the donors that were treated with 0.5 mg/kg L-Thyroxine and 10 mg/kg PTU; R_PTU, the recipients that were colonized by cecal microbiota from PTU-treated donors; R_YJT, the recipients that were colonized by cecal microbiota from YJT+PTU -treated donors; R_T4, the recipients that were colonized by cecal microbiota from L-thyroxin+PTU-treated donors; R_Con, the rats that were colonized with cecal microbiota from control donors.

Supplementary Table 4 The relative abundence of every taxa at the phylum level in the donors and recipient rats.

Taxonomy	Total	D_Con	D_PTU	D_YJT	D_T4	R_Con	R_PTU	R_YJT	R_T4
Bacteroidota	59.0%	42.3%	53.9%	57.6%	48.8%	62.6%	71.6%	69.7%	65.7%
Cyanobacteria	3.5%	2.1%	2.3%	3.1%	1.8%	2.8%	4.9%	6.2%	4.7%
Deferribacterota	0.2%	0.6%	0.1%	0.1%	0.1%	0.2%	0.0%	0.2%	0.1%
Desulfobacterota	0.0%	0.0%	0.0%	0.0%	0.0%	0.0%	0.0%	0.0%	0.0%
Elusimicrobiota	0.1%	0.0%	0.2%	0.4%	0.0%	0.0%	0.4%	0.0%	0.0%
Firmicutes	36.4%	54.4%	42.7%	37.8%	48.7%	33.7%	22.4%	22.9%	28.7%
Proteobacteria	0.8%	0.4%	0.9%	1.0%	0.5%	0.7%	0.8%	1.0%	0.9%

D_Con, the donors of control group that just received phosphate buffered saline (PBS); D_PTU, the donors that received 10 mg/kg Propylthiouracil (PTU); D_YJT (D_YJT+PTU), the donors that received 2.1g/kg YJT and 10 mg/kg PTU; D_T4 (D_T4+PTU), the donors that were treated with 0.5 mg/kg L-Thyroxine and 10 mg/kg PTU; R_PTU, the recipients that were colonized by cecal microbiota from PTU-treated donors; R_YJT, the recipients that were colonized by cecal microbiota from YJT+PTU-treated donors; R_T4, the recipients that were colonized by cecal microbiota from L-thyroxin+PTU-treated donors; R_Con, the rats that were colonized with cecal microbiota from control donors.

Supplementary Table 5 The relative abundence of every taxa at the genus level in the donors and recipient rats.

Taxonomy	Total	D_Con	D_PTU	D_YJT	D_T4	R_Con	R_PTU	R_YJT	R_T4
Bacteroides	21.1%	9.9%	17.2%	22.5%	15.5%	25.6%	26.2%	23.1%	28.6%
Muribaculaceae	31.3%	29.1%	27.5%	30.9%	29.7%	30.9%	30.5%	41.8%	29.8%
Prevotellaceae_UCG-001	2.2%	1.2%	4.3%	1.3%	0.6%	1.3%	7.9%	0.5%	0.7%
Alistipes	3.7%	2.1%	3.7%	2.6%	2.9%	4.4%	5.6%	3.5%	4.8%
Rikenellaceae_RC9_gut_group	0.2%	0.0%	0.3%	0.2%	0.1%	0.1%	0.1%	0.4%	0.5%
Gastranaerophilales	3.5%	2.1%	2.3%	3.1%	1.8%	2.8%	4.9%	6.2%	4.7%
Mucispirillum	0.2%	0.6%	0.1%	0.1%	0.1%	0.2%	0.0%	0.2%	0.1%
Elusimicrobium	0.1%	0.0%	0.2%	0.4%	0.0%	0.0%	0.4%	0.0%	0.0%
Anaeroplasma	4.8%	7.2%	9.4%	9.6%	7.9%	0.0%	4.1%	0.0%	0.2%
Turicibacter	0.3%	0.0%	0.5%	0.4%	0.0%	0.2%	0.9%	0.1%	0.4%
Lactobacillus	0.6%	0.5%	0.7%	0.6%	0.2%	0.3%	0.7%	0.8%	0.5%
RF39	0.1%	0.1%	0.3%	0.2%	0.1%	0.1%	0.2%	0.2%	0.0%
Clostridia_UCG-014	0.9%	0.5%	1.5%	1.0%	0.7%	0.2%	0.6%	0.3%	2.2%
Clostridia_vadinBB60_group	1.0%	0.9%	0.6%	0.7%	1.1%	1.4%	0.7%	1.9%	1.0%
Clostridium_sensu_stricto_1	0.1%	0.0%	0.1%	0.1%	0.0%	0.0%	0.4%	0.1%	0.0%
A2	0.1%	0.1%	0.1%	0.1%	0.3%	0.0%	0.0%	0.0%	0.0%

ASF356	0.0%	0.1%	0.1%	0.0%	0.1%	0.0%	0.0%	0.0%	0.0%
Anaerostipes	0.4%	0.5%	1.3%	0.0%	0.0%	0.5%	0.7%	0.1%	0.3%
Blautia	0.1%	0.0%	0.0%	0.0%	0.0%	0.2%	0.2%	0.0%	0.0%
Eisenbergiella	0.1%	0.0%	0.1%	0.0%	0.0%	0.2%	0.5%	0.0%	0.3%
Frisingicoccus	0.1%	0.0%	0.0%	0.0%	0.0%	0.1%	0.1%	0.1%	0.0%
Lachnoclostridium	0.2%	0.4%	0.1%	0.2%	0.5%	0.1%	0.0%	0.1%	0.0%
Lachnospiraceae_NK4A136_group	7.6%	12.1%	8.1%	4.2%	9.0%	12.1%	3.0%	6.0%	6.0%
Lachnospiraceae_UCG-001	0.1%	0.1%	0.1%	0.2%	0.4%	0.0%	0.0%	0.0%	0.0%
Lachnospiraceae_UCG-006	0.1%	0.2%	0.1%	0.2%	0.1%	0.1%	0.0%	0.1%	0.1%
Lachnospiraceae_UCG-008	0.3%	0.0%	0.1%	0.1%	0.0%	0.1%	1.6%	0.1%	0.3%
Marvinbryantia	0.0%	0.0%	0.0%	0.0%	0.0%	0.0%	0.1%	0.0%	0.0%
Roseburia	0.3%	0.9%	0.2%	0.4%	0.6%	0.0%	0.0%	0.1%	0.1%
[Eubacterium]_ventriosum_group	0.0%	0.1%	0.1%	0.0%	0.0%	0.0%	0.0%	0.0%	0.0%
[Eubacterium]_xylanophilum_group	0.3%	0.4%	0.4%	0.3%	0.2%	0.4%	0.0%	0.3%	0.3%
Monoglobus	0.2%	0.0%	0.1%	0.1%	0.0%	0.7%	0.0%	0.1%	0.2%
Butyricicoccus	0.1%	0.1%	0.0%	0.1%	0.1%	0.2%	0.0%	0.0%	0.1%
UCG-009	0.0%	0.0%	0.0%	0.0%	0.0%	0.0%	0.0%	0.0%	0.0%
Colidextribacter	0.9%	1.1%	0.7%	1.1%	1.2%	0.9%	0.7%	0.7%	0.6%
Intestinimonas	0.1%	0.0%	0.0%	0.0%	0.4%	0.0%	0.0%	0.0%	0.0%
NK4A214_group	0.0%	0.0%	0.1%	0.1%	0.1%	0.0%	0.0%	0.0%	0.0%
Oscillibacter	0.5%	0.9%	0.8%	0.5%	0.7%	0.3%	0.3%	0.3%	0.4%
UCG-003	0.2%	0.2%	0.1%	0.2%	0.6%	0.4%	0.1%	0.2%	0.2%
UCG-005	0.5%	0.2%	1.1%	1.2%	0.1%	0.5%	0.1%	0.2%	0.3%
Anaerotruncus	0.0%	0.0%	0.0%	0.0%	0.1%	0.0%	0.0%	0.0%	0.0%
Harryflintia	0.0%	0.0%	0.1%	0.0%	0.0%	0.0%	0.0%	0.0%	0.0%
Incertae_Sedis	0.1%	0.1%	0.0%	0.1%	0.1%	0.2%	0.0%	0.1%	0.1%
Ruminococcaceae	0.0%	0.2%	0.0%	0.0%	0.1%	0.0%	0.0%	0.0%	0.0%
Ruminococcus	1.7%	1.0%	3.6%	2.5%	0.6%	1.9%	1.0%	1.5%	1.4%
[Eubacterium]_siraeum_group	0.2%	0.5%	0.0%	0.1%	0.5%	0.0%	0.1%	0.0%	0.1%
UCG-010	0.0%	0.0%	0.1%	0.0%	0.0%	0.0%	0.0%	0.0%	0.0%
[Eubacterium]_coprostanoligenes_group	0.5%	0.3%	0.5%	0.6%	0.3%	0.7%	0.1%	0.7%	0.5%
Peptococcus	0.1%	0.1%	0.0%	0.1%	0.1%	0.0%	0.0%	0.0%	0.0%
Anaerovorax	0.0%	0.1%	0.1%	0.1%	0.0%	0.0%	0.1%	0.1%	0.0%

Romboutsia	0.4%	0.1%	0.6%	0.5%	0.2%	0.5%	0.5%	0.3%	0.4%
Parasutterella	0.4%	0.2%	0.2%	0.3%	0.4%	0.5%	0.4%	0.5%	0.3%
Other	14.1%	25.4%	12.0%	12.3%	21.8%	11.8%	7.0%	9.2%	14.1%

D_Con, the donors of control group that just received phosphate buffered saline (PBS); D_PTU, the donors that received 10 mg/kg Propylthiouracil (PTU); D_YJT (D_YJT+PTU), the donors that received 2.1g/kg YJT and 10 mg/kg PTU; D_T4 (D_T4+PTU), the donors that were treated with 0.5 mg/kg L-Thyroxine and 10 mg/kg PTU; R_PTU, the recipients that were colonized by cecal microbiota from PTU-treated donors; R_YJT, the recipients that were colonized by cecal microbiota from YJT+PTU-treated donors; R_T4, the recipients that were colonized by cecal microbiota from L-thyroxin+PTU-treated donors; R_Con, the rats that were colonized with cecal microbiota from control donors.

Supplementary Table 6. Sequences of the primers for RT-qPCR

Gene	Forward primer (5'-3')	Reverse primer (5'-3')
Adrb3	GGCAGAACTCACCGCTCAAC	TCCAGAAGTCAGGCTCCTTGC
Cidea	CAATGGAGACCGCCAGGGAC	AAACCCGGAAAGGACGAGCA
Claudin-2	TCGAGAAAGAACAGCTCCGTTT	CTTGTCTTTTGGCTGCGGCT
Dio1	TTCCTGGCGCTCTATGACTCG	GACACGTGCACCACACTGGA
Dio2	ACAGAAGTGCAACGTCTGGGA	CCAGGTTTACCCTGTGGCGT
Fabp4	AATGTGCGACGCCTTTGTGG	TACCGGCCACTTTCCTGGTG
FFAR2	CTGTGGGGATCCACAGCCCT	AACTGTGCCAGTCTGGGGTC
FFAR3	CCTACAGCCAGGGGACCAAC	CAGCCATTTCCAGTCGCACG
FXR	AGGAAGTGCAGAGAGATGGGA	GCTTGGTCGTGGAGGTCACT
GAPDH	TTCTAGAGACAGCCGCAT	TGGTAACCAGGTGTCCGA
GLP-1R	AGGTAGTCTTTGCCCATGCC	CCCCTAGGCAGGTTACTCCT
GPR65	TTGGAAGGGTGGCCAGATGT	TACTGTGCCGGACCCTTGAG
HDAC4	GGGGGAGCAGCATCATGGTT	CTGCGCAAACTCGAAGTCCC
HTR1F	ACCTGTCGGCTATAGCGTTG	CTGCTTTGCGTTCTCGAGTG
IL-15	TGTGGGCATCTGAATCCACT	TGCTGCCTCTCGGAATACTCA
IL-6	TCATTCTGTCTCGAGCCCACC	CTGGCTGGAAGTCTCTTGCG
NF-κB	ACCACTGTCAACAGATGGCCC	ACCTTTGCAGGCCCCACATA
Nod2	GAGGAGCTCTGACTCCAAGCA	TGTCCAAGCGTCCCACTGAC
PCNA	TTTGAGGCACGCCTGATCCA	GCAGCGGTATGTGTCGAAGC
Prdm16	ACTTCGAGCTGCGAGAGTCC	GCAGCTCTCCTGGGATGACA
PGC-1α	TGGGACATGTGCAGCCAAGA	GGCAAAGAGGCTGGTCCTCA

Pglyrp1	CCGCAATGTGCAGCTTTACCA	TGTGGTCACCCTTGATGGTCC
Pglyrp2	AGCAGATTAATTCTCATGCCCCT	TGACCATGGGTAAGGAGGAGG
PPAR-α	GCTGGGTCCTCTGGTTGTCC	AGTTCACAGGGAAGGCAGGG
PPAR-γ	TCCTGTTGACCCAGAGCATGG	TGATTCCGAAGTTGGTGGGC
TGR5	TCCACTTGGCCCCCAACTTT	GCACCAGGACTCCAGTGGTT
Th	CGTCTCAGAGCAGGATGCCA	CGATGAGACTCTGTCGCCGT
TLR4	TTTACAGAGGGGCAACCGCT	GGCCGTGAGAAAGCATGACG
TNFα	GCGCTCCCCAAAAAGATGGG	GGACCGATCACCCCGAAGTT
Tph2	TGGATTCAGCGGTGCCAGAA	TCCTCGCTTTTGCTAGCGGT
Trpv1	CGGTTCTGGAGGTGATCGCT	GCCTTCCACAGGCCGATAGT
Trpv3	GGTGATCTCAAAGCAAGGGCTG	GGAGTGGGCATTCATTGCTGG
Trpv4	TCATTAACGAGGACCCCGGC	CACCACTGAGGACCAGCGAT
ZO-1	GTGCAAAGAGATGAGCGGGC	GGTCGGGAGATCGTGACTGG
UCP1	ACACTGTGGAAAGGGACGAC	GCAAAACCCGGCGACAAGAG

Supplementary Table 7. Barcode sequences and primers for Exp. 1

Sample ID	Barcode Sequence	Forward Primer Sequence	Reverse Primer Sequence	Type
A33	TAATCGCG	CCTACGGGNGGCWGCAG	GACTACHVGGGTATCTAATCC	Con
A36	TACCGCAA	CCTACGGGNGGCWGCAG	GACTACHVGGGTATCTAATCC	Con
B31	CTACACGA	CCTACGGGNGGCWGCAG	GACTACHVGGGTATCTAATCC	Con
C01	AATAGGCG	CCTACGGGNGGCWGCAG	GACTACHVGGGTATCTAATCC	PTU
C02	ACACAGAG	CCTACGGGNGGCWGCAG	GACTACHVGGGTATCTAATCC	PTU
C04	ACACCTGA	CCTACGGGNGGCWGCAG	GACTACHVGGGTATCTAATCC	PTU
C05	ACAGACAG	CCTACGGGNGGCWGCAG	GACTACHVGGGTATCTAATCC	PTU
C06	ACAGCAGA	CCTACGGGNGGCWGCAG	GACTACHVGGGTATCTAATCC	PTU
C08	TAATGCCG	CCTACGGGNGGCWGCAG	GACTACHVGGGTATCTAATCC	PTU
C09	TACCAACG	CCTACGGGNGGCWGCAG	GACTACHVGGGTATCTAATCC	PTU
C10	TACCGCAA	CCTACGGGNGGCWGCAG	GACTACHVGGGTATCTAATCC	PTU
C13	ACCACATG	CCTACGGGNGGCWGCAG	GACTACHVGGGTATCTAATCC	YJT+PTU
C14	ACCACTAG	CCTACGGGNGGCWGCAG	GACTACHVGGGTATCTAATCC	YJT+PTU
C15	ACCATGCA	CCTACGGGNGGCWGCAG	GACTACHVGGGTATCTAATCC	YJT+PTU
C16	ACCTAGCA	CCTACGGGNGGCWGCAG	GACTACHVGGGTATCTAATCC	YJT+PTU

C17	ACCTCAAG	CCTACGGGNGGCWGCAG	GACTACHVGGGTATCTAATCC	YJT+PTU
C21	ACGAGATG	CCTACGGGNGGCWGCAG	GACTACHVGGGTATCTAATCC	T4+PTU
C23	TGAGACAG	CCTACGGGNGGCWGCAG	GACTACHVGGGTATCTAATCC	T4+PTU
C24	ACGTGTTG	CCTACGGGNGGCWGCAG	GACTACHVGGGTATCTAATCC	T4+PTU
C27	ACTGCACA	CCTACGGGNGGCWGCAG	GACTACHVGGGTATCTAATCC	T4+PTU
C28	AATAGCGG	CCTACGGGNGGCWGCAG	GACTACHVGGGTATCTAATCC	T4+PTU
C29	ACACAGAG	CCTACGGGNGGCWGCAG	GACTACHVGGGTATCTAATCC	T4+PTU
C32	ACACCTGA	CCTACGGGNGGCWGCAG	GACTACHVGGGTATCTAATCC	Con
C34	TTGCAACG	CCTACGGGNGGCWGCAG	GACTACHVGGGTATCTAATCC	Con
C35	TTGCGGTA	CCTACGGGNGGCWGCAG	GACTACHVGGGTATCTAATCC	Con
C39	TTGGCGTA	CCTACGGGNGGCWGCAG	GACTACHVGGGTATCTAATCC	Con

Supplementary Table 8. Barcode sequences and primers for Exp. 2

Sample ID	Barcode Sequence	Forward Primer Sequence	Reverse Primer Sequence	Type
S01	AACCAAGG	CCTACGGGNGGCWGCAG	GACTACHVGGGTATCTAATCC	CMT^{PTU}
S02	AACCTACG	CCTACGGGNGGCWGCAG	GACTACHVGGGTATCTAATCC	CMT^{PTU}
S03	AACGGCAA	CCTACGGGNGGCWGCAG	GACTACHVGGGTATCTAATCC	CMT^{PTU}
S04	AAGCCGAA	CCTACGGGNGGCWGCAG	GACTACHVGGGTATCTAATCC	CMT^{PTU}
S06	ACGACTTG	CCTACGGGNGGCWGCAG	GACTACHVGGGTATCTAATCC	CMT^{YJT}
S07	AAGGTTGG	CCTACGGGNGGCWGCAG	GACTACHVGGGTATCTAATCC	CMT^{YJT}
S08	AATAGCGG	CCTACGGGNGGCWGCAG	GACTACHVGGGTATCTAATCC	CMT^{YJT}
S09	AATAGGCG	CCTACGGGNGGCWGCAG	GACTACHVGGGTATCTAATCC	CMT^{YJT}
S10	ACACAGAG	CCTACGGGNGGCWGCAG	GACTACHVGGGTATCTAATCC	CMT^{YJT}
S11	CGAACCTA	CCTACGGGNGGCWGCAG	GACTACHVGGGTATCTAATCC	Vehicle
S12	ACGTGTTG	CCTACGGGNGGCWGCAG	GACTACHVGGGTATCTAATCC	Vehicle
S13	ACAGCAGA	CCTACGGGNGGCWGCAG	GACTACHVGGGTATCTAATCC	Vehicle
S14	CCATATGG	CCTACGGGNGGCWGCAG	GACTACHVGGGTATCTAATCC	Vehicle
S15	CCATGCAA	CCTACGGGNGGCWGCAG	GACTACHVGGGTATCTAATCC	Vehicle
S16	ACCACATG	CCTACGGGNGGCWGCAG	GACTACHVGGGTATCTAATCC	CMT^{T4}
S17	CGAACGAA	CCTACGGGNGGCWGCAG	GACTACHVGGGTATCTAATCC	CMT^{T4}
S18	ACCAGTTG	CCTACGGGNGGCWGCAG	GACTACHVGGGTATCTAATCC	CMT^{T4}
S19	ACCATGCA	CCTACGGGNGGCWGCAG	GACTACHVGGGTATCTAATCC	CMT^{T4}

S20	ACCTAGCA	CCTACGGGNGGCWGCAG	GACTACHVGGGTATCTAATCC	CMT^{T4}
S21	ACACCTGA	CCTACGGGNGGCWGCAG	GACTACHVGGGTATCTAATCC	CMT^{Con}
S22	ACCTCTTG	CCTACGGGNGGCWGCAG	GACTACHVGGGTATCTAATCC	CMT^{Con}
S23	AGACACAG	CCTACGGGNGGCWGCAG	GACTACHVGGGTATCTAATCC	CMT^{Con}
S24	CCAATACG	CCTACGGGNGGCWGCAG	GACTACHVGGGTATCTAATCC	CMT^{Con}
S25	ACGACAAG	CCTACGGGNGGCWGCAG	GACTACHVGGGTATCTAATCC	CMT^{Con}

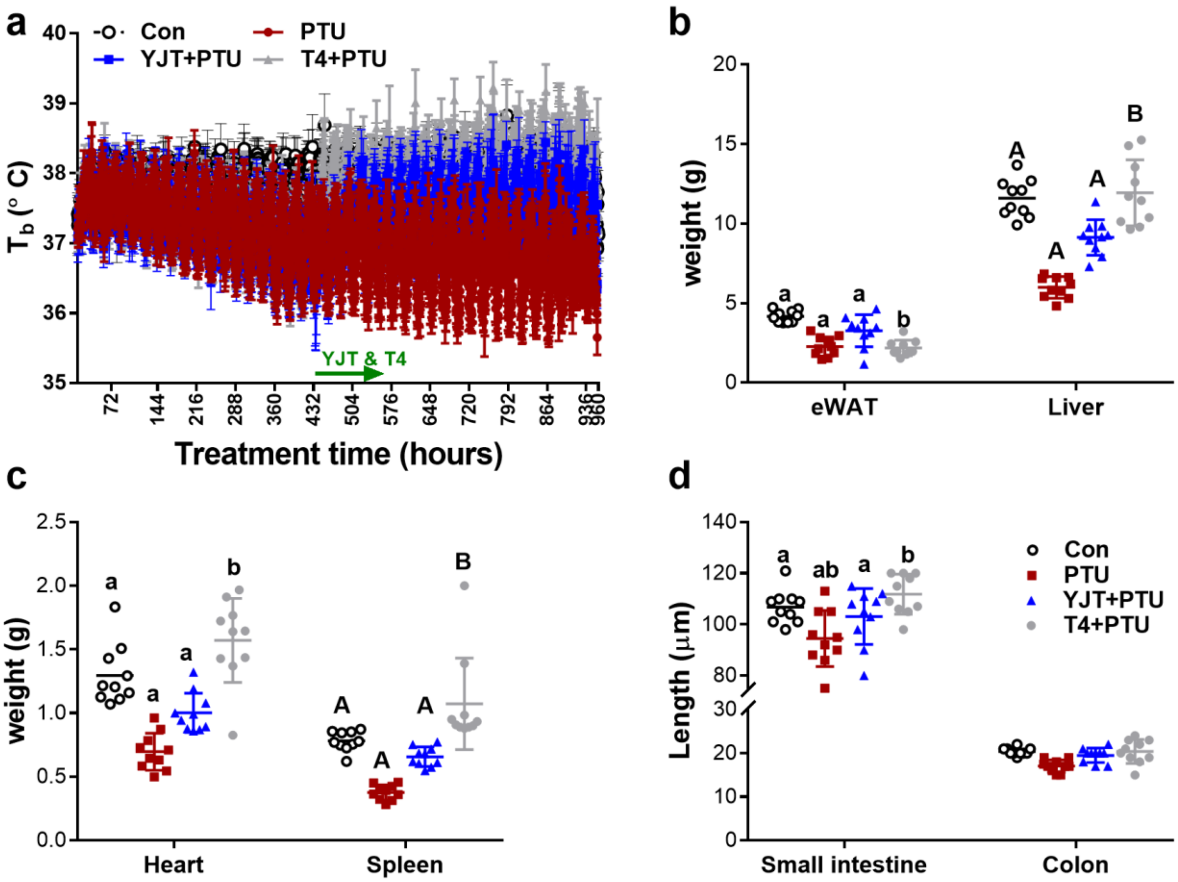

Supplementary Figure 1. Core body temperature (T_b) and organ masses in different treatment groups

(a) T_b was monitored every half an hour. (b-d) Organ morphology in the different treatment groups. Data are presented as means ± SEM (n = 6-7 per group). Different letters above columns indicate significant differences. Con, the control group that just received saline; PTU, the rats that received 10 mg/kg Propylthiouracil (PTU); YJT+PTU, the rats that received 2.1g/kg YJT and 10 mg/kg PTU; T4+PTU, the rats that were treated with 0.5 mg/kg L-Thyroxine and 10 mg/kg PTU.

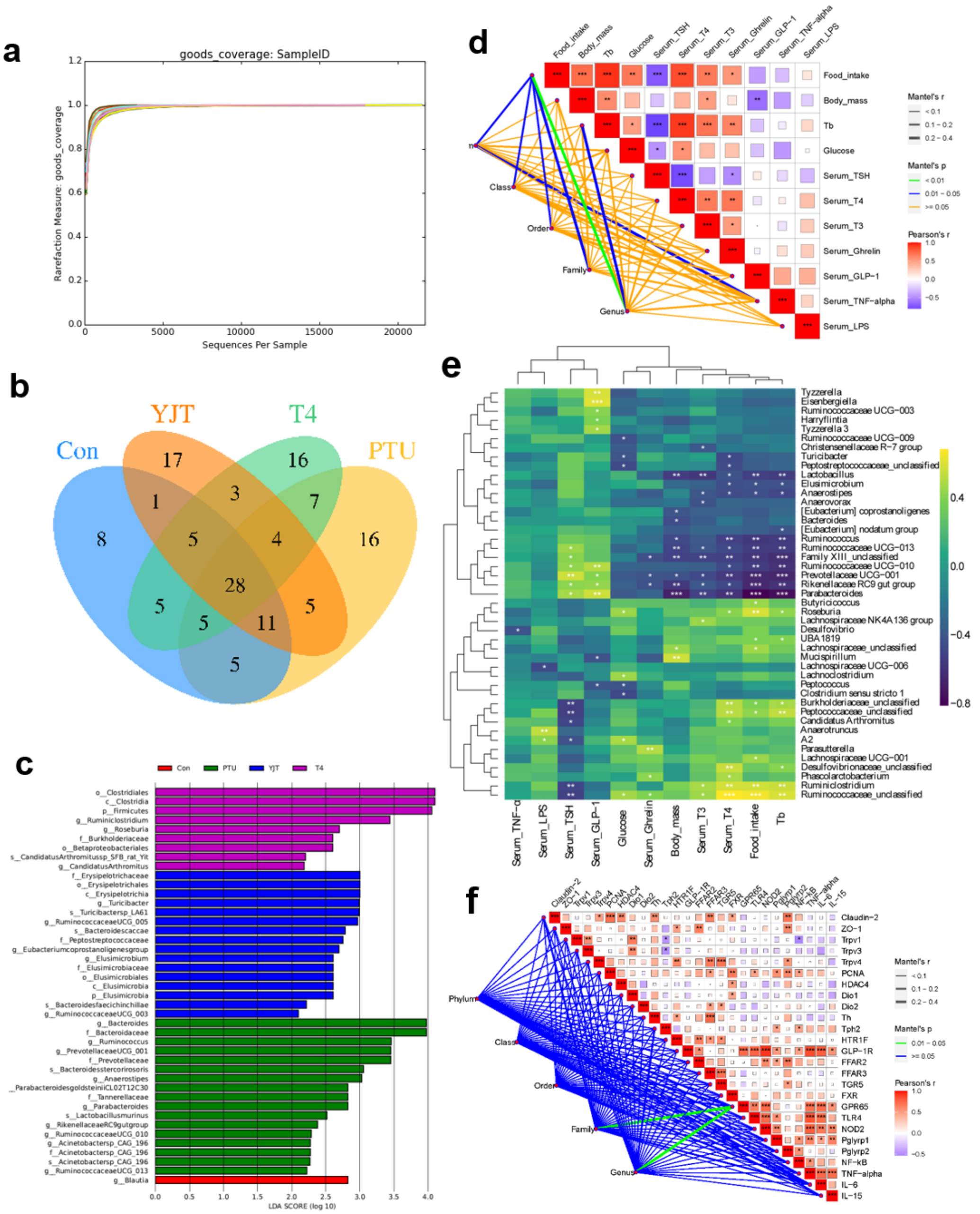

Supplementary Figure 2. Fecal microbiota and their correlation with host variables.

(a) The rarefaction curve of Goods coverage for all the fecal samples. (b) The Venn diagram displayed the core amplicon sequence variants (ASVs) across 85% of samples in each group. (c) Differential bacterial biomarkers selected by LEfSe analysis with LDA score > 2 in the fecal microbiota community for each group. (d) Mantel test and Pearson's correlations between differential metabolic phenotypes, serum metabolites and bacteria taxa (Mantel's P＜0.05 was indicated by blue lines; and P＜0.01 by green lines). (e) Heatmap of correlation between bacterial genera, host metabolic phenotypes and serum metabolites. (f) Mantel test and Pearson's correlations (*P < 0.05, **P < 0.01, and ***P < 0.001) between differential thermogenesis and inflammatory markers in the small inestine and bacteria taxa (Mantel's P＜0.05 was indicated by green lines). Con, the control group that

just received saline; PTU, the rats that received 10 mg/kg Propylthiouracil (PTU); YJT+PTU, the rats that received 2.1g/kg YJT and 10 mg/kg PTU; T4+PTU, the rats that were treated with 0.5 mg/kg L-Thyroxine and 10 mg/kg PTU.

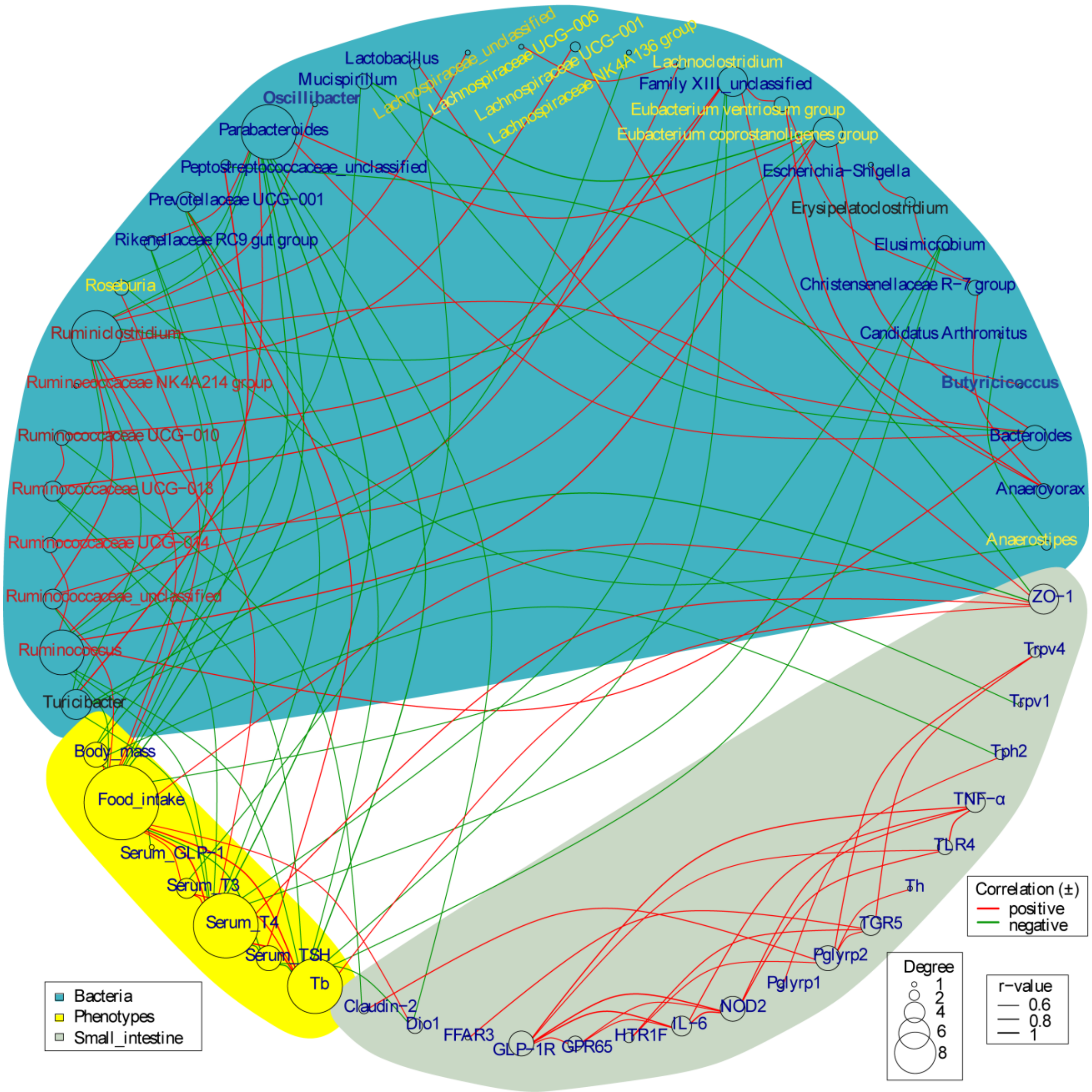

Supplementary Figure 3. A co-occurrence network constructed from the relative abundances of differential bacterial genera and host phenotypes. The differential bacterial genera mainly generated three covarying enriched units. They are the genus *Parabacterioides* belonging to f_Tannerellaceae (p_Bacteroidota), seven genera (in red font) belonging to f_Ruminococcaceae (p_Firmicutes), and nine genera (in yellow font) belonging to f_Lachnospiraceae (p_Firmicutes). The genera from the same p_Firmicutes are usually positively correlated with each other; whereas they are negatively correlated with the genus from p_Bacteroidota. The degree of these nodes was indicated by different sizes of circles which represents the relative abundances of these differential variables. Edges between nodes indicate Pearson's positive (red) or negative (green) correlation and edge thickness displays range of r value (r > 0.6).

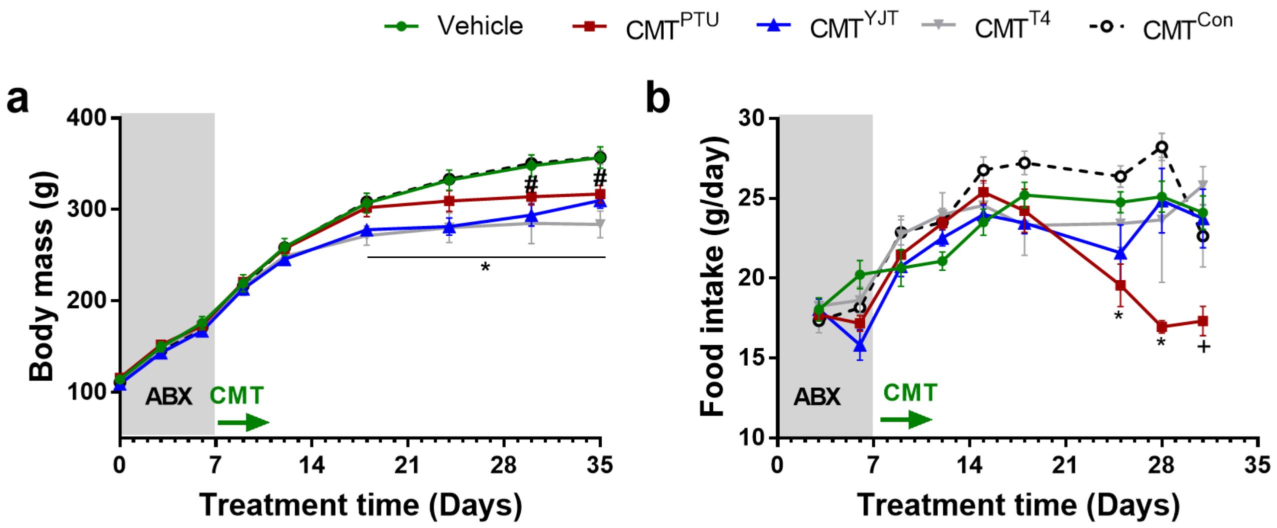

Supplementary Figure 4. Effect of cecal microbiota transfer (CMT) on body mass and food intake.

(a) Body mass; *P < 0.05 CMT^{YJT} and CMT^{T4} vs Vehicle and CMT^{con}; $^{\#}P$ < 0.05 CMT^{PTU} vs Vehicle and CMT^{con}. (b) Food intake; *P < 0.05 versus Vehicle and CMT^{con}; ^{+}P < 0.05 versus Vehicle, CMT^{YJT}, and CMT^{T4}. Two-way ANOVA followed by post hoc LSD test. Data are presented as means ± SEM. ABX, antibiotics treatment for senven days; Vehicle, the control rats which were orally gavaged with phosphate buffered saline; CMT^{PTU}, the rats that were colonized by donors of the PTU-treated group; CMT^{YJT}, the rats that were colonized by microbiota from the YJT+PTU-treated animals; CMT^{T4}, the rats that were colonized with donors of the L-thyroxin+PTU-treated group; CMT^{Con}, the rats that were colonized with microbiota from the control donors.

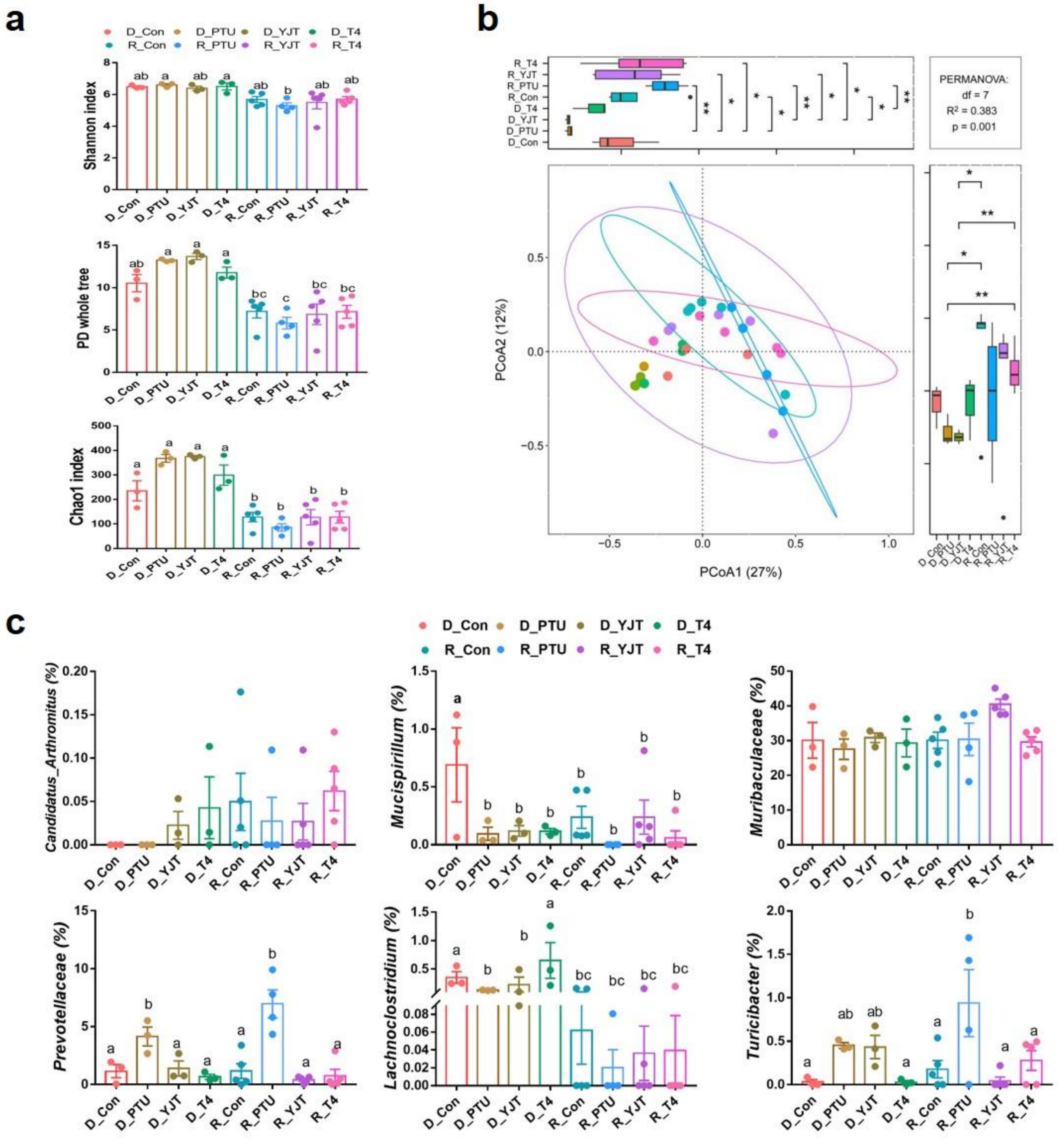

Supplementary Figure 5. The comparison of microbiota diversity and composition between the donors and recipients.

(a) The α diversity of microbiota community in the donors and recipients. (b) The β diversity indicated by principal coordinate analyses (PCoA) based on the Bray-Curtis dissimilarity in the fecal microbiota to analyze and visualize similarities and differences among samples. The boxplots on the top and right display the indices of Bray-Curtis dissimilarity along the PCo1 and PCo2 axis for subjects in each group, and statistical differences were determined by permutational multivariate analysis of variance (PERMANOVA, $*P < 0.05$, and $**P < 0.01$). (c) The relative abundance of different bacteria in the donors and recipients at the genus level. Different letters above columns indicate significant differences. D_Con, the donors of control group that just received

phosphate buffered saline (PBS); D_PTU, the donors that received 10 mg/kg Propylthiouracil (PTU); D_YJT, the donors that received 2.1g/kg YJT and 10 mg/kg PTU; D_T4, the donors that were treated with 0.5 mg/kg L-Thyroxine and 10 mg/kg PTU; R_PTU, the recipients that were colonized by cecal microbiota from PTU-treated donors; R_YJT, the recipients that were colonized by cecal microbiota from YJT+PTU-treated donors; R_T4, the recipients that were colonized by cecal microbiota from L-thyroxin+PTU-treated donors; R_Con, the rats that were colonized with cecal microbiota from control donors.

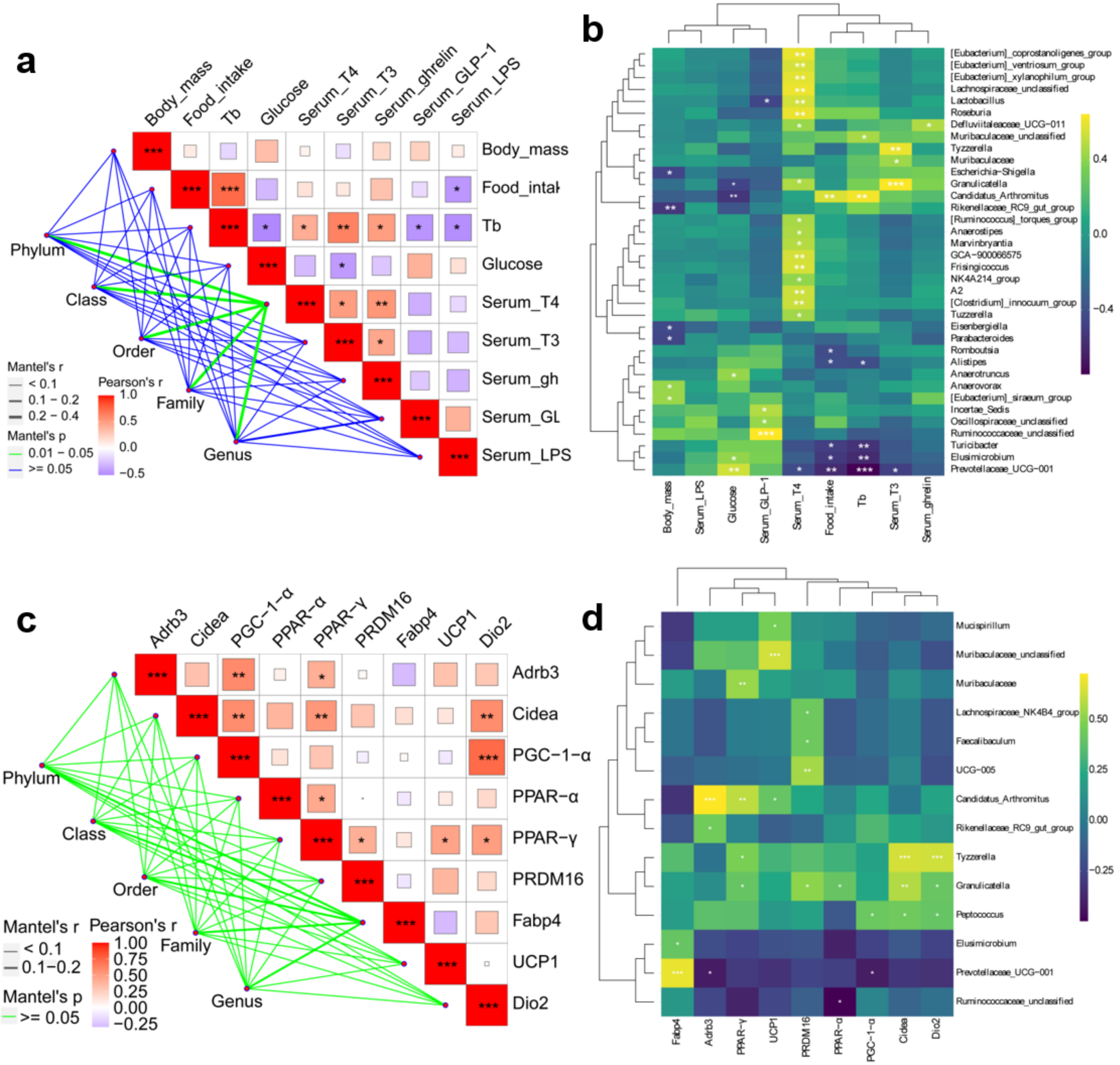

Supplementary Figure 6. Correlation between metabolic phenotypes, serum metabolites, metabolism markers and bacteria taxa in Exp. 2.

(a) Mantel test and Pearson's correlations between differential metabolic phenotypes, serum metabolites and bacteria taxa (Mantel's $P < 0.05$ was indicated by blue lines; and $P < 0.01$ by green lines). (b) Heatmap of Pearson's correlation between bacterial genera, host metabolic phenotypes and serum metabolites. (c) Mantel test and Pearson's correlations between differential thermogenesis markers in the brown adipose tissue (BAT) and bacteria taxa (Mantel's $P > 0.05$ was indicated by green lines). (d) Heatmap of Pearson's correlation between bacterial genera and host thermogenesis markers in BAT. Pearson's correlation, *$P < 0.05$,**$P < 0.01$, ***$P < 0.001$.

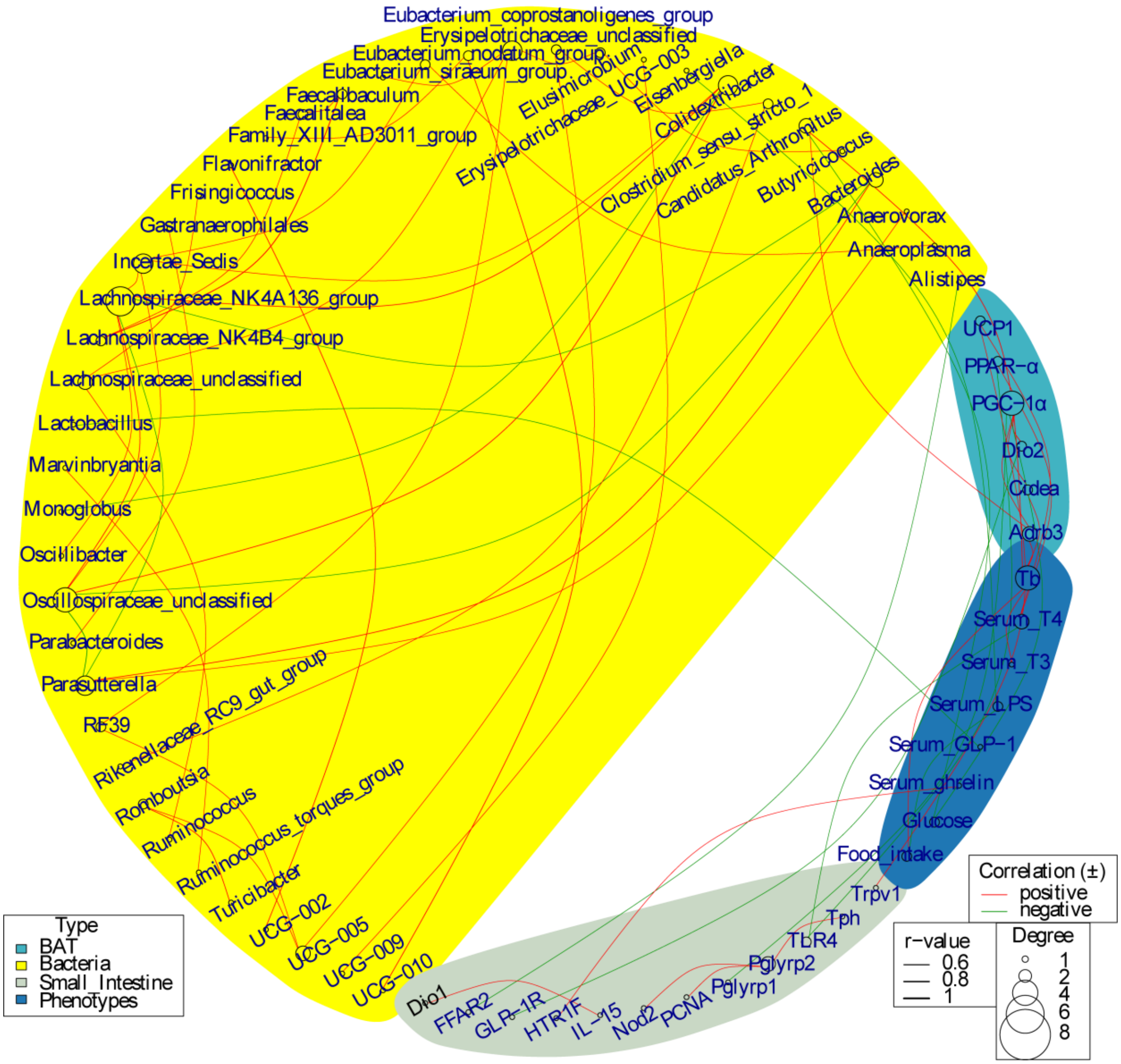

Supplementary Figure 7. A co-occurrence network constructed from the relative abundances of differential bacterial genera, host phenotypes and gene expression in BAT and small intestine. The degree of these nodes was indicated by different sizes of circles which represents the relative abundances of these differential variables. Edges between nodes indicate Pearson's positive (red) or negative (green) correlation and edge thickness displays range of r value (r > 0.6). BAT, brown adipose tissue.

Supplementary codes

#Part I: Basic data analysis using QIIME2

#Step1: Input Data, Demultiplex reads

qiime tools import --type 'SampleData[PairedEndSequencesWithQuality]' --input-path input.tsv --output-path demux.qza --input-format PairedEndFastqManifestPhred33V2

#Visualization

qiime demux summarize --i-data demux.qza --o-visualization demux.qzv

#Step2: Denoising and clustering. Denoising the reads into amplicon sequence variants

qiime dada2 denoise-paired --i-demultiplexed-seqs demux.qza --p-trunc-len-f 0 --p-trunc-len-r 0 --p-n-threads 24 --o-table table.qza --o-representative-sequences rep_set.qza --o-denoising-stats stats.qza

#Visualization

qiime metadata tabulate --m-input-file stats.qza --o-visualization stats.qzv

qiime feature-table summarize --i-table table.qza --m-sample-metadata-file Mapping.txt --o-visualization table.qzv

qiime feature-table tabulate-seqs --i-data rep_set.qza --o-visualization rep_set.qzv

#Step3: Filter low frequency and features sample

#Filter out sample of less than 2000 sequences

qiime feature-table filter-samples --i-table table.qza --p-min-frequency 2000 --o-filtered-table table_fil_1.qza

#Filter out rare ASVs

qiime feature-table filter-features --i-table table_fil_1.qza --p-min-frequency 2 --p-min-samples 1 --o-filtered-table table_fil_2.qza

#Filter rep set

qiime feature-table filter-seqs --i-data rep_set.qza --i-table table_fil_2.qza --o-filtered-data rep_set_2.qza

#Step4: Assign taxonomy to ASVs

qiime feature-classifier classify-sklearn --i-reads rep_set_2.qza --i-classifier silva-132-99-nb-classifier.qza --p-n-jobs 6 --o-classification taxonomy.qza

qiime metadata tabulate --m-input-file taxonomy.qza --o-visualization taxonomy.qzv

qiime tools export --input-path taxonomy.qza --output-path ./

#Filter out contaminant and unclassified ASVs

qiime taxa filter-table --i-table table_fil_2.qza --i-taxonomy taxonomy.qza --p-include D_1__ --p-exclude mitochondria,chloroplast,Eukaryota --o-filtered-table table_filter.qza

qiime feature-table summarize --i-table table_filter.qza --o-visualization table_filter.qzv

#Subset and summarize filtered table

qiime feature-table filter-seqs --i-data rep_set_2.qza --i-table table_filter.qza --o-filtered-data rep_set_filter.qza

qiime feature-table tabulate-seqs --i-data rep_set_filter.qza --o-visualization rep_set_filter.qzv

qiime tools export --input-path rep_set_filter.qza --output-path TemporaryFolder/basic_analysis

#Step5: Build tree

qiime phylogeny align-to-tree-mafft-fasttree --i-sequences rep_set_filter.qza --o-alignment aligned-rep-seqs.qza --o-masked-alignment masked-aligned-rep-seqs.qza --o-tree unrooted-tree.qza --o-rooted-tree rooted-tree.qza

#Step6: Trans QZA format of AVS to BIOM

qiime tools export --input-path table_filter.qza --output-path ./

biom add-metadata -i feature-table.biom -o feature-table_tax.biom --observation-metadata-fp taxonomy.tsv --sc-separated taxonomy --observation-header ASVID,taxonomy

biom summarize-table -i feature-table_tax.biom -o feature-table_tax_summary.txt

#Step7-1: Generate stacked barchart of taxa relative abundances

qiime taxa barplot --i-table table_filter.qza --i-taxonomy taxonomy.qza --m-metadata-file $mappingFile --o-visualization taxonomy_barplot.qzv

qiime tools export --input-path taxonomy_barplot.qzv --output-path Taxonomy_Barplot

#Step7-2: Generate stacked barchart of taxa relative abundances of Groups

qiime feature-table group --i-table table_filter.qza --p-axis sample --p-mode mean-ceiling --m-metadata-file $mappingFile --m-metadata-column Group --o-grouped-table table_filter_Group.qza

qiime taxa barplot --i-table table_filter_Group.qza --i-taxonomy taxonomy.qza --m-metadata-file group.tsv --o-visualization taxonomy_barplot_filter_Group.qzv

qiime tools export --input-path taxonomy_barplot_filter_Group.qzv --output-path Taxonomy_Barplot_Group

#Step8: Alpha and beta diversity analysis

qiime diversity core-metrics-phylogenetic --i-phylogeny rooted-tree.qza --i-table table_filter.qza --p-sampling-depth 20000 --m-metadata-file Mapping.txt --output-dir core-metrics-results

#Step9. Pathway analysis using PICRUST2 (method 2)

picrust2_pipeline.py -s dna-sequences.fasta -i feature-table_tax.biom -o pathway -p 24 --in_traits COG,EC,KO,PFAM,TIGRFAM

pathway_pipeline.py -i pathway/KO_metagenome_out/pred_metagenome_unstrat.tsv.gz -o pathway/KEGG_pathways_out --no_regroup --map /home/wangjf/software/Tools/Miniconda3/envs/qiime2-2021.2/lib/python3.6/site-packages/picrust2/default_files/pathway_mapfiles/KEGG_pathways_to_KO.tsv

#TSV to BIOM

biom convert -i pathway/KEGG_pathways_out/path_abun_unstrat.tsv.gz -o KEGG.biom --to-hdf5

biom add-metadata -i KEGG.biom -o pathway/KEGG_pathways_out/KEGG.biom --observation-metadata-fp KEGG.xls --sc-separated taxonomy --observation-header KOID,taxonomy

biom convert -i pathway/COG_metagenome_out/pred_metagenome_unstrat.tsv.gz -o COG.biom --to-hdf5

biom add-metadata -i COG.biom -o pathway/COG_metagenome_out/COG.biom --observation-metadata-fp COG.xls --sc-separated taxonomy --observation-header COGID,taxonomy

Part II: Plotting and statistical analysis using third-party software

#Step10. Beta diversity and plots

beta_diversity_through_plots.py -i feature-table_tax.biom -m Mapping.txt -t rep_set.tre -e 20000 -o BetaDiversity

#Comparing Categories using ANOSIM

compare_categories.py --method anosim -i bray_curtis_dm.txt -m Mapping.txt -c Group -o Group_bray_curtis_anosim -n 999

compare_categories.py --method anosim -i unweighted_unifrac_dm.txt -m Mapping.txt -c Group -o Group_unweighted_unifrac_anosim -n 999

compare_categories.py --method anosim -i weighted_unifrac_dm.txt -m Mapping.txt -c Group -o Group_weighted_unifrac_anosim -n 999

#Comparing Categories using PERMANOVA

compare_categories.py --method permanova -i bray_curtis_dm.txt -m Mapping.txt -c Group -o Group_bray_curtis_permanova -n 999

compare_categories.py --method permanova -i unweighted_unifrac_dm.txt -m Mapping.txt -c Group -o Group_unweighted_unifrac_permanova -n 999

compare_categories.py --method permanova -i weighted_unifrac_dm.txt -m Mapping.txt -c Group -o Group_weighted_unifrac_permanova -n 999

#Comparing Categories using ADONIS

compare_categories.py --method adonis -i bray_curtis_dm.txt -m Mapping.txt -c Group -o Group_bray_curtis_adonis_out -n 999

compare_categories.py --method adonis -i unweighted_unifrac_dm.txt -m Mapping.txt -c Group -o Group_unweighted_unifrac_adonis_out -n 999

compare_categories.py --method adonis -i weighted_unifrac_dm.txt -m Mapping.txt -c Group -o Group_weighted_unifrac_adonis_out -n 999

#Plotting PCoA

#Bray_Curtis

PCoA.py -i bray_curtis_pc.txt -m Mapping.txt -d 3 -o Group_bray_curtis_3d.pdf -b Group -c col.txt --figsize 8 8 -s 30 --ggplot2_style

PCoA.py -i bray_curtis_pc.txt -m Mapping.txt -d 2 -o Group_bray_curtis_2d.pdf -b Group -c col.txt --figsize 8 8 -s 30 --ggplot2_style

#Step11. Cluster Analysis

mkdir ClusterAnalysis

metaphlan_hclust_heatmap.py --in ClusterAnalysis/Taxa.xls --out ClusterAnalysis/phyla_abundance_heatmap.png -c bbcry --top 10 --minv 0.0001 -s log --tax_lev p

metaphlan_hclust_heatmap.py --in ClusterAnalysis/Taxa.xls --out ClusterAnalysis/class_abundance_heatmap.png -c bbcry --top 20 --minv 0.0001 -s log --tax_lev c

metaphlan_hclust_heatmap.py --in ClusterAnalysis/Taxa.xls --out ClusterAnalysis/orders_abundance_heatmap.png -c bbcry --top 30 --minv 0.0001 -s log --tax_lev o

metaphlan_hclust_heatmap.py --in ClusterAnalysis/Taxa.xls --out ClusterAnalysis/families_abundance_heatmap.png -c bbcry --top 40 --minv 0.0001 -s log --tax_lev f

metaphlan_hclust_heatmap.py --in ClusterAnalysis/Taxa.xls --out ClusterAnalysis/genera_abundance_heatmap.png -c bbcry --top 50 --minv 0.0001 -s log --tax_lev g

metaphlan_hclust_heatmap.py --in ClusterAnalysis/Taxa.xls --out ClusterAnalysis/species_abundance_heatmap.png -c bbcry --top 50 --minv 0.0001 -s log --tax_lev s

#Step12. High-Dimensional biomarker discovery and explanation using LEfSe

mkdir Biomarker

format_input.py Taxa.xls Group.in -c 1 -s 2 -u 3 -o 1000000

run_lefse.py Group.in Group.out -l 2 -y 0

plot_res.py Group.out Group.lefse_biomarker.png --feature_font_size 10 --width 10 --dpi 300 --format png --left_space 0.3

plot_cladogram.py Group.out Group.lefse_biomarkers_cladogram.png --class_legend_font_size 6.5 --dpi 300 --format png

plot_features.py Group.in Group.out Group.biomarkers.zip --archive zip --format png

#Step13. Function Analysis

mkdir Function

mkdir Function/KEGG

categorize_by_function.py -i pathway/KEGG_pathways_out/KEGG.biom -c KEGG_Pathways -l 3 -o Function/KEGG/predicted_metagenomes.L3.biom

categorize_by_function.py -i pathway/KEGG_pathways_out/KEGG.biom -c KEGG_Pathways -l 3 -o Function/KEGG/predicted_metagenomes.L3.txt -f

categorize_by_function.py -i pathway/KEGG_pathways_out/KEGG.biom -c KEGG_Pathways -l 2 -o Function/KEGG/predicted_metagenomes.L2.biom

categorize_by_function.py -i pathway/KEGG_pathways_out/KEGG.biom -c KEGG_Pathways -l 2 -o Function/KEGG/predicted_metagenomes.L2.txt -f

categorize_by_function.py -i pathway/KEGG_pathways_out/KEGG.biom -c KEGG_Pathways -l 1 -o Function/KEGG/predicted_metagenomes.L1.biom

categorize_by_function.py -i pathway/KEGG_pathways_out/KEGG.biom -c KEGG_Pathways -l 1 -o Function/KEGG/predicted_metagenomes.L1.txt -f

mkdir Function/COG

categorize_by_function.py -i Function/metagenome_predictions_cog.biom -c COG_Category -l 2 -o Function/COG/predicted_metagenomes.L2.biom

categorize_by_function.py -i Function/metagenome_predictions_cog.biom -c COG_Category -l 2 -o Function/COG/predicted_metagenomes.L2.txt -f

categorize_by_function.py -i Function/metagenome_predictions_cog.biom -c COG_Category -l 1 -o Function/COG/predicted_metagenomes.L1.biom

categorize_by_function.py -i Function/metagenome_predictions_cog.biom -c COG_Category -l 1 -o Function/COG/predicted_metagenomes.L1.txt –f

#Step14. R code for PCoA Plot

```
rm(list=ls())

pacman::p_load(tidyverse,ggrepel,vegan,ape,ggsignif,patchwork,multcomp)

data <- read.csv("ASV_table.txt", header = T,check.names = F,sep="	",row.names = 1) %>% t()

data[is.na(data)] <- 0

pcoa <- vegdist(data,method = "bray") %>% pcoa(correction = "none", rn = NULL)

groups <- read.table("groups.txt",sep = "	",header = T) %>% as.list()

PC1 = pcoa$vectors[,1]

PC2 = pcoa$vectors[,2]

pcoadata <- data.frame(rownames(pcoa$vectors),PC1,PC2,groups$Type)

colnames(pcoadata) <-c("sample","PC1","PC2","group")

yf <- pcoadata

yd1 <- yf %>% group_by(group) %>% summarise(Max = max(PC1))

yd2 <- yf %>% group_by(group) %>% summarise(Max = max(PC2))

yd1$Max <- yd1$Max + max(yd1$Max)*0.1

yd2$Max <- yd2$Max + max(yd2$Max)*0.1

pcoadata$group = as.factor(pcoadata$group)

res1 <- aov(PC1~group,data = pcoadata) %>% glht(linfct=mcp(group="Tukey")) %>% cld(alpah=0.05)

res2 <- aov(PC2~group,data = pcoadata) %>% glht(linfct=mcp(group="Tukey")) %>% cld(alpah=0.05)

test <- data.frame(PC1 = res1$mcletters$Letters,PC2 = res2$mcletters$Letters, yd1 = yd1$Max,yd2 =
yd2$Max,group = yd1$group)

p1 <- ggplot(pcoadata, aes(PC1, PC2)) +
geom_point(aes(colour=group,fill=group),size=4)+stat_ellipse(aes(color = group),level = 0.95, show.legend =
F)+labs(x=(floor(pcoa$values$Relative_eig[1]*100)) %>% paste0("PC1 ( ", ., "%",
" )"),y=(floor(pcoa$values$Relative_eig[2]*100)) %>% paste0("PC2 ( ", ., "%", " )")) +
theme(text=element_text(size=12))+geom_vline(aes(xintercept =
0),linetype="dotted")+geom_hline(aes(yintercept = 0),linetype="dotted")+ theme(panel.background =
element_rect(fill='white', colour='black'),axis.title.x=element_text(colour='black',
```

```
size=12),axis.title.y=element_text(colour='black', size=12),
axis.text=element_text(colour='black',size=12),legend.title=element_blank(),legend.position = "none")

p2 <- ggplot(pcoadata,aes(group,PC1)) + geom_boxplot(aes(fill = group)) + geom_signif(comparisons =
list(c("BD_PTU","CD_YJT"),c("BD_PTU","DD_T4"),c("BD_PTU","AD_Con"),c("BD_PTU","FR_PTU"),c("
BD_PTU","GR_YJT"),c("BD_PTU","HR_T4"),c("BD_PTU","ER_Con"),c("CD_YJT","DD_T4"),c("CD_YJT
","AD_Con"),c("CD_YJT","FR_PTU"),c("CD_YJT","GR_YJT"),c("CD_YJT","HR_T4"),c("CD_YJT","ER_C
on"),c("DD_T4","AD_Con"),c("DD_T4","FR_PTU"),c("DD_T4","GR_YJT"),c("DD_T4","HR_T4"),c("DD_T
4","ER_Con"),c("AD_Con","FR_PTU"),c("AD_Con","GR_YJT"),c("AD_Con","HR_T4"),c("AD_Con","ER_
Con"),c("FR_PTU","GR_YJT"),c("FR_PTU","HR_T4"),c("FR_PTU","ER_Con"),c("GR_YJT","HR_T4"),c("
GR_YJT","ER_Con"),c("HR_T4","ER_Con")),map_signif_level=T,textsize=4,test=t.test,step_increase=0.2) +
theme(panel.background = element_rect(fill='white',colour='black'))+ theme(axis.ticks.length = unit(0.4,'lines'),
axis.ticks = element_line(color='black'),axis.line = element_line(colour = 'black'),
axis.title.x=element_blank(),axis.title.y=element_blank(),axis.text.y=element_text(colour='black',size=10,face =
'plain'),axis.text.x=element_blank(),legend.position = 'none')+coord_flip()

p3 <- ggplot(pcoadata,aes(group,PC2)) + geom_boxplot(aes(fill = group)) + geom_signif(comparisons =
list(c("BD_PTU","CD_YJT"),c("BD_PTU","DD_T4"),c("BD_PTU","AD_Con"),c("BD_PTU","FR_PTU"),c("
BD_PTU","GR_YJT"),c("BD_PTU","HR_T4"),c("BD_PTU","ER_Con"),c("CD_YJT","DD_T4"),c("CD_YJT
","AD_Con"),c("CD_YJT","FR_PTU"),c("CD_YJT","GR_YJT"),c("CD_YJT","HR_T4"),c("CD_YJT","ER_C
on"),c("DD_T4","AD_Con"),c("DD_T4","FR_PTU"),c("DD_T4","GR_YJT"),c("DD_T4","HR_T4"),c("DD_T
4","ER_Con"),c("AD_Con","FR_PTU"),c("AD_Con","GR_YJT"),c("AD_Con","HR_T4"),c("AD_Con","ER_
Con"),c("FR_PTU","GR_YJT"),c("FR_PTU","HR_T4"),c("FR_PTU","ER_Con"),c("GR_YJT","HR_T4"),c("
GR_YJT","ER_Con"),c("HR_T4","ER_Con")),map_signif_level=T,textsize=4,test=t.test,step_increase=0.2) +
theme(panel.background = element_rect(fill='white',colour='black'))+ theme(axis.ticks.length = unit(0.4,'lines'),
axis.ticks = element_line(color='black'),axis.line = element_line(colour = 'black'),
axis.title.x=element_blank(),axis.title.y=element_blank(),axis.text.x=element_text(colour='black',size=10,angle
= 45,vjust = 0.5,hjust = 0.5,face = 'plain'),axis.text.y=element_blank(),legend.position = 'none')

otu.adonis=adonis(data~group,data = pcoadata,distance = "bray")

p4 <- ggplot()+geom_text(aes(x = -0.5,y = 0.6,label = paste("PERMANOVA:

df = ",otu.adonis$aov.tab$Df[1],"

R2 = ",round(otu.adonis$aov.tab$R2[1],4),"\np-value = ",otu.adonis$aov.tab$`Pr(>F)`[1],sep = "")),size = 4)
+theme_bw() + xlab(NULL) + ylab(NULL) + theme(panel.grid=element_blank(), axis.title =
element_blank(),axis.line = element_blank(),axis.ticks = element_blank(),axis.text = element_blank())
```

```
p <- p2+p4+p1+p3 + plot_layout(heights = c(1,4),widths = c(4,1),ncol = 2,nrow = 2)

ggsave(p, filename = "PCoA_Type_bray.pdf", width=8, height=8, dpi = 300)


#Step15. R code for Taxa-Circle Plot by Type

rm(list=ls())

pacman::p_load(tidyverse,circlize)

df <- read.delim("ASV_table.txt",check.names = F) %>% dplyr::rename(otu=`ID`) %>% separate(taxonomy, into=c("domain","phylum","class","order","family","genus"),sep="; ") %>% select(C01:S25,genus) %>% drop_na() %>% rowwise() %>% mutate(sum = sum(across(where(is.numeric)))) %>% arrange(desc(sum)) %>% head(15) %>% select(-sum) %>% pivot_longer(-genus) %>% mutate_at(vars(-name,-value),~str_split(.,"__",simplify=TRUE)[,2]) %>% filter(genus != "uncultured") %>% dplyr::rename(SampleID=name) %>% left_join(.,read.delim("groups.txt"),by="SampleID") %>% select(-SampleID) %>% select(genus,Type,value)

pdf("Taxa-Circle_Plot_by_Type.pdf", width=12, height=10)

circos.par(canvas.xlim=c(-1,1),canvas.ylim=c(-1,1.2),start.degree = 0)

set.seed(1234)

chordDiagram(df, link.sort = FALSE, link.decreasing = TRUE, transparency = 0.1, annotationTrack = "grid", preAllocateTracks = list(track.height = .1))

for(si in get.all.sector.index()) {

  xlim = get.cell.meta.data("xlim",sector.index = si,track.index = 1)

  ylim = get.cell.meta.data("ylim",sector.index = si,track.index = 1)

  circos.text(mean(xlim), ylim[1],labels = si,sector.index = si, track.index = 1, facing = "clockwise", cex=0.8, adj=c(0,.5), niceFacing = T)

}

circos.clear()

dev.off()
```

#Step16. R code for Heatmap

```
rm(list=ls())

library(pheatmap)

rt=read.table("Phylum.txt",header=T,sep="	",row.names=1,check.names=F)

ann=read.table("groups.txt",header=T,sep=" ",row.names=1,check.names=F)

p1 <- pheatmap(rt, annotation=ann, cluster_cols = T,  color = colorRampPalette(c("blue", "white", "red"))(50), show_colnames = T, scale="row", fontsize = 8, fontsize_row=6, fontsize_col=6)

pdf("Type_Heatmap_1.pdf",width=8,height=2.5)

p1

dev.off()

p2 <- pheatmap(rt, annotation=ann, cluster_cols = F,  color = colorRampPalette(c("blue", "white", "red"))(50), show_colnames = T, scale="row", fontsize = 8, fontsize_row=6, fontsize_col=6)

pdf("Type_Heatmap_2.pdf",width=8,height=2.5)

p2

dev.off()
```

#Step17. R script to draw Heatmap for the correlation between ASVs and physiological measurements.

```
library(ggplot2)

library(reshape2)

library(plyr)

library(scales)

library(RColorBrewer)

dat <- read.table("genus.tab",header=T,sep="\t")

dat$ID <- with(dat,reorder(ID, Taxonomy))

dat.m <- melt(dat)

dat.m$value2<-cut(dat.m$value,breaks=c(-Inf, 0.001, 0.01, 0.05),label=c("***", "**", "*"))
```

```
p <- ggplot(dat.m, aes(dat.m$variable,dat.m$ID)) + geom_tile(aes(fill = value),colour = "white",show.legend=T)
+ scale_fill_gradient(low = "red",high = "white") + theme_bw() + xlab("") + ylab("ASV ID") +
theme(axis.text.x=element_text(angle=-90,hjust=1,size=10)) +
theme(axis.text.y=element_text(angle=0,hjust=1,size=8,colour=dat.m$Taxonomy)) + labs(fill="FDR")

p <- p + geom_text(aes(label=dat.m$value),size=5,na.rm=TRUE,show.legend=T,nudge_x=0,nudge_y=-0.3)

ggsave(file="Genus_HeatMap.pdf",width=6,height=10,plot=p)
```
